# Exploring the Impact of the HOMO–LUMO Gap on Molecular Thermoelectric Properties: A Comparative Study of Conjugated Aromatic, Quinoidal, and Donor–Acceptor Core Systems

**DOI:** 10.1021/acsomega.3c09760

**Published:** 2024-02-05

**Authors:** Nickel Blankevoort, Pablo Bastante, Ross J. Davidson, Rebecca J. Salthouse, Abdalghani H. S. Daaoub, Pilar Cea, Santiago Martin Solans, Andrei S. Batsanov, Sara Sangtarash, Martin R. Bryce, Nicolas Agrait, Hatef Sadeghi

**Affiliations:** † Device Modelling Group, School of Engineering, University of Warwick, Coventry CV4 7AL, U.K.; ‡ Departamento de Física de la Materia Condensada C-III, Universidad Autónoma de Madrid, E-28049 Madrid, Spain; § Department of Chemistry, Durham University, Durham DH1 3LE, U.K.; ∥ Instituto de Nanociencia y Materiales de Aragón (INMA), CSIC−Universidad de Zaragoza, 50009 Zaragoza, Spain; ⊥ Departamento de Química Física, Universidad de Zaragoza, 50009 Zaragoza, Spain; # Laboratorio de Microscopias Avanzadas (LMA), Universidad de Zaragoza, 50018 Zaragoza, Spain; ∇ Condensed Matter Physics Center (IFIMAC) and Instituto Universitario de Ciencia de Materiales “Nicolás Cabrera”, Universidad Autónoma de Madrid, 28049 Madrid, Spain

## Abstract

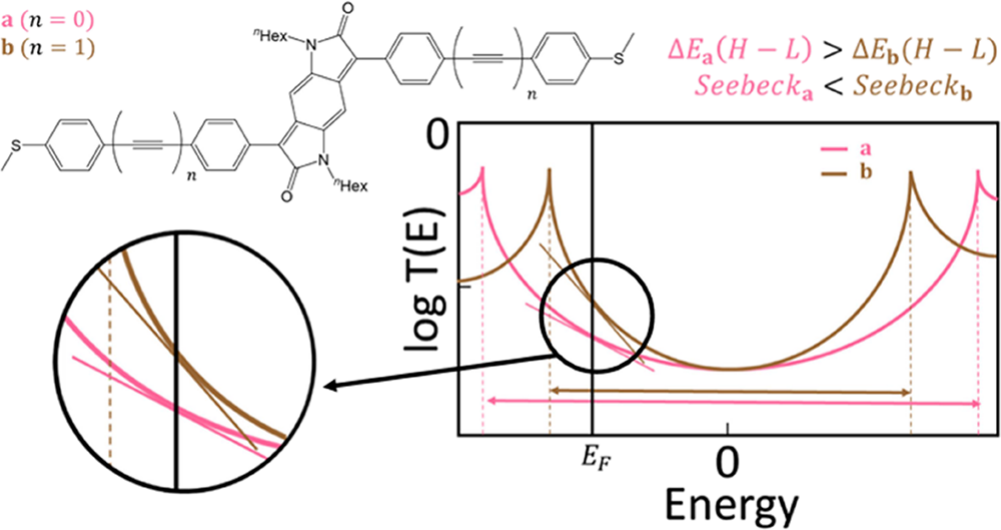

Thermoelectric materials have garnered significant interest for their potential to efficiently convert waste heat into electrical energy at room temperature without moving parts or harmful emissions. This study investigated the impact of the HOMO–LUMO (H-L) gap on the thermoelectric properties of three distinct classes of organic compounds: conjugated aromatics (isoindigos (IIGs)), quinoidal molecules (benzodipyrrolidones (BDPs)), and donor–acceptor systems (bis(pyrrol-2-yl)squaraines (BPSs)). These compounds were chosen for their structural simplicity and linear π-conjugated conductance paths, which promote high electrical conductance and minimize complications from quantum interference. Single-molecule thermoelectric measurements revealed that despite their low H-L gaps, the Seebeck coefficients of these compounds remain low. The alignment of the frontier orbitals relative to the Fermi energy was found to play a crucial role in determining the Seebeck coefficients, as exemplified by the BDP compounds. Theoretical calculations support these findings and suggest that anchor group selection could further enhance the thermoelectric behavior of these types of molecules.

## Introduction

Thermoelectric materials have the potential to revolutionize energy conversion by directly converting waste heat into useful electrical energy at room temperature without the need for any moving parts or environmentally harmful emissions.^1^ They can be used in a variety of applications, ranging from power generation in automobiles and industrial processes via the Seebeck effect to cooling of electronics and other devices via the Peltier effect.^2−4^ Current inorganic thermoelectric materials, such as bismuth telluride (Bi_2_Te_3_),^5^ lead telluride (PbTe),^6^ and silicon germanium (SiGe),^7^ have been widely studied and utilized, but their applications are restricted due to toxicity and limited availability, as well as a generally low conversion efficiency at room temperature. Also, in most semiconductors, the increase in conductance is accompanied by a decrease of the Seebeck coefficient, which is not desirable and makes improving the thermoelectric efficiency a difficult task.^8−14^

**Figure 1:**
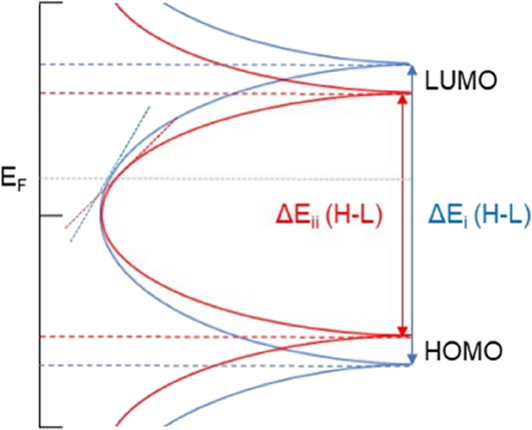
Example of a transmission function that determines the probability that an electron of a given energy will tunnel through a molecular junction where Δ*E*_*i*_(*H* – *L*) > Δ*E*_*ii*_(*H* – *L*). Increasing the transmission gradient around the Fermi energy by reducing the band gap can benefit the Seebeck coefficient.

These limitations have led to increased research on organic alternatives, which are more environmentally sustainable and potentially more efficient.^15,16^ Improving the thermoelectric efficiency of such materials is achieved by simultaneously increasing the electrical conductance (*G*) and Seebeck coefficient (*S*) while keeping the thermal conductance low.^17,18^ Given that the Seebeck coefficient^19^ relates to the gradient of the transmission curve (see eq 1), one of the most effective strategies to improve molecular thermopower is to employ quantum interference (QI), i.e., introducing a sharp resonance feature^20−25^ within the highest occupied molecular orbital (HOMO) and lowest unoccupied molecular orbital (LUMO) gap. However, this is often achieved using destructive QI, which results in reduced electrical conductance. An alternate approach that has yet to be directly examined is the impact of the HOMO–LUMO (H-L) gap and changes in the position of resonances.^26−28^ In principle, the reduction of the H-L gap for a given molecule with a nonmid-gap Fermi energy (*E*_F_)^29−33^ should result in an increased transmission gradient, i.e., Seebeck coefficient enhancement without any decrease in electrical conductance; see Figure 1.
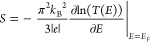
1where *k*_B_ is the Boltzmann constant, *e* is the electron charge, and *T*(*E*) is the transmission probability as a function of energy, *E*.

Such a phenomenon has been used to explain the Seebeck coefficient increase for oligophenylene molecules as their length is extended.^34,35^ However, the impacts were small in these examples and it was not until the work of Dell et al. using a donor–acceptor (D-A) system comprising thiophene D and oxidized oligothiophenes as A units that increasing the molecules’ length, which reduced the H-L gap from 2.1 to 1.4 eV, was shown to result in a significant Seebeck coefficient change from +7.3 to −22.1 μV/K.^36^ A similarly high Seebeck coefficient (ca. 15.5 μV/K) has also been observed for a 4,7-di(thiophen-2-yl)benzothiadiazole-based system.^37^ However, both of the previous examples are D-A-based compounds; therefore, this question arises: is this a feature intrinsic to such systems or is it universal to all low-H-L-gap compounds?

To investigate this, we synthesized and measured the conductance and thermoelectric behavior of three different groups of compounds with an H-L gap <2.2 eV. The new molecules comprise an aromatic, a quinoidal (singlet diradical), and a D-A core system, based on isoindigo (IIG), benzodipyrrolidone (BDP), and bis(pyrrol-2-yl)squaraine (BPS), respectively. These structures were chosen due to their relatively simple structures and π-conjugated “linear” conductance paths, which should promote comparatively high electrical conductance and avoid complications from QI features. Herein, their conductance and thermoelectric properties were examined experimentally and theoretically at the single-molecule level in the tunneling regime.^38^ For clarity, the IIG, BDP, and BPS derivatives are denoted as “**1**”, “**2**”, and “**3**”, respectively, and their short and long variants are denoted as “**a**” and “**b**”, respectively.

## Results and Discussion

### Synthesis

**Figure 2:**
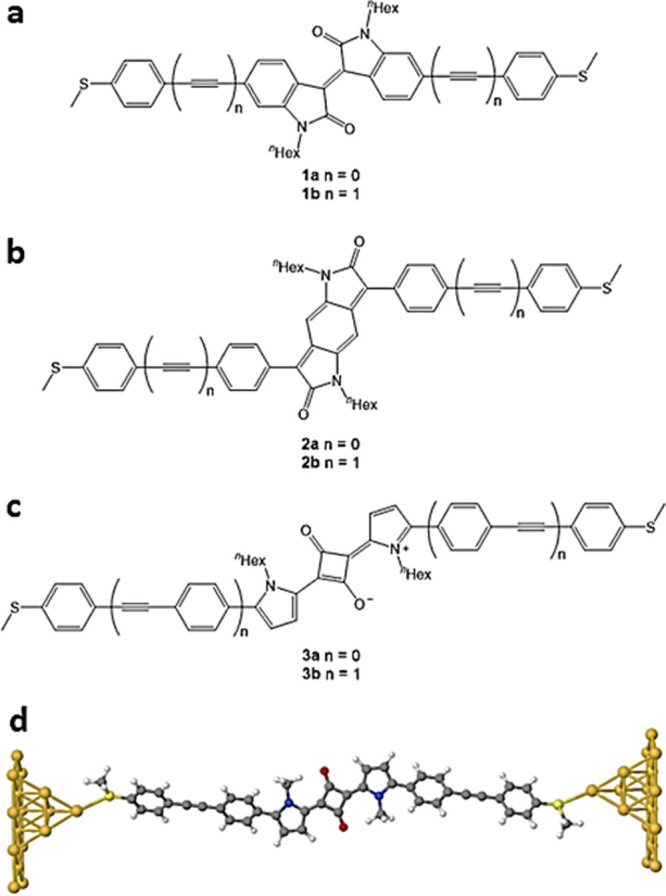
Overview of the molecular structures of (a) **1a** and **1b**, (b) **2a** and **2b**, and (c) **3a** and **3b**. (d) Structure **3b** (with Me representing an *n*-Hex chain for clarity) in a junction with gold electrodes, assumed to be the most likely geometry of the junction with both thiomethyl groups acting as anchors.

Both the IGG-based (**1a** and **1b**) (Figure 2a) and BDP-based compounds (**2a** and **2b**) (Figure 2b) were synthesized using a dibromo-substituted building block (6,6′-dibromo-1,1′-dihexyl-[3,3′-biindolinylidene]-2,2′-dione^39^ or 3,7-bis(4-bromophenyl)-1,5-dihexylpyrrolo[2,3]indole-2,6-dione, respectively) via Stille coupling with tributyl(4-(methylthio)phenyl)stannane or Sonogashira coupling with (4-ethynylphenyl)(methyl)sulfane to produce thiomethyl-anchored compounds. It is noteworthy that when Suzuki–Miyaura coupling was attempted with (4-(methylthio)phenyl)boronic acid and either of the dibromo-building blocks, there was no evidence of a reaction with the building blocks. The BPS compounds (**3a** and **3b**) (Figure 2c) were synthesized using an adaptation of So et al.’s approach,^40^ whereby substituted 1-hexyl-pyrrole (1-hexyl-2-(4-(methylthio)phenyl)-*1H*-pyrrole or 1-hexyl-2-(4-((4-(methylthio)phenyl)ethynyl)phenyl)-1*H*-pyrrole) was heated with squaric acid to produce the respective BPS. All of the compounds were characterized using NMR spectroscopy, mass spectrometry, and elemental analysis, and compounds **1b** and **2b** were also characterized by single-crystal X-ray crystallography (see the Supporting Information (SI), Section A).

### Experimental Methods and Results

**Figure 3:**
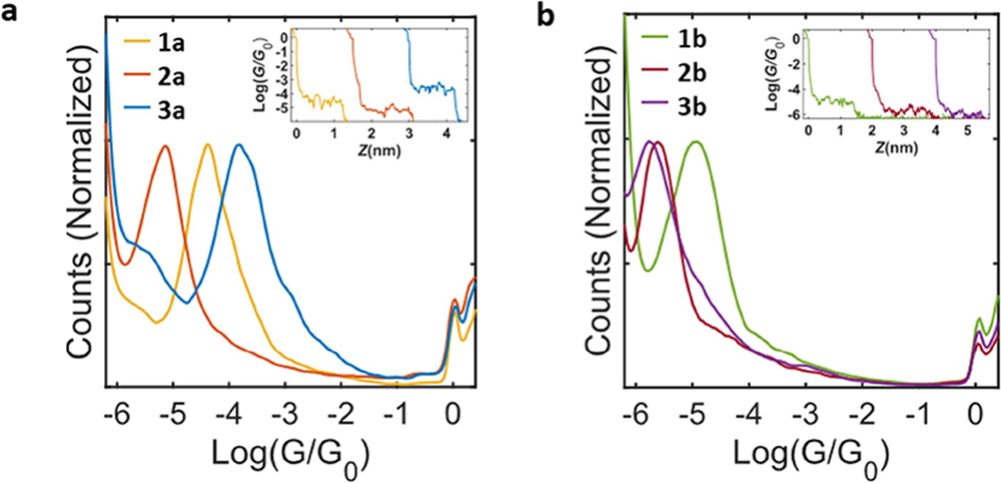
(a,b) Conductance histograms for short “**a**” and long “**b**” counterparts of compounds **1**, **2**, and **3** and (inset) examples of individual traces showing a molecular plateau around the mean value of the conductance distribution for each compound.

All molecules were trapped in a gold|single molecule|gold junction, as illustrated in Figure 2d for **3b**, for the measurement of electrical conductance and the Seebeck coefficient. These junctions were formed by using a homebuilt scanning tunneling microscope (STM) under ambient conditions. The molecules were deposited on a preannealed gold surface (Arrandee) from a ∼1 mM solution in DCM. Then, a mechanically cut 0.25 mm diameter gold wire (Goodfellow) was used as the tip and indented to the surface. While retracting, the junction is reduced to the atomic level until the gold contact breaks, where statistically a single molecule is contacted between the tip and the sample. These were connected electrically to apply a bias voltage and measure the current, hence acting as electrodes. The procedure was performed thousands of times at a constant bias voltage of 100 mV to measure the current when the junction breaks to obtain the current vs distance traces (insets in Figure 3a,b). When a molecule is contacted, there is a plateau between the single-atom plateau at a conductance of *G*_0_ = 7.75 × 10^–5^ S and the noise level of the open circuit. These traces were used to build the conductance histograms shown in Figure 3a,b, where the counts create a distribution around the most probable conductance value.

**Figure 4:**
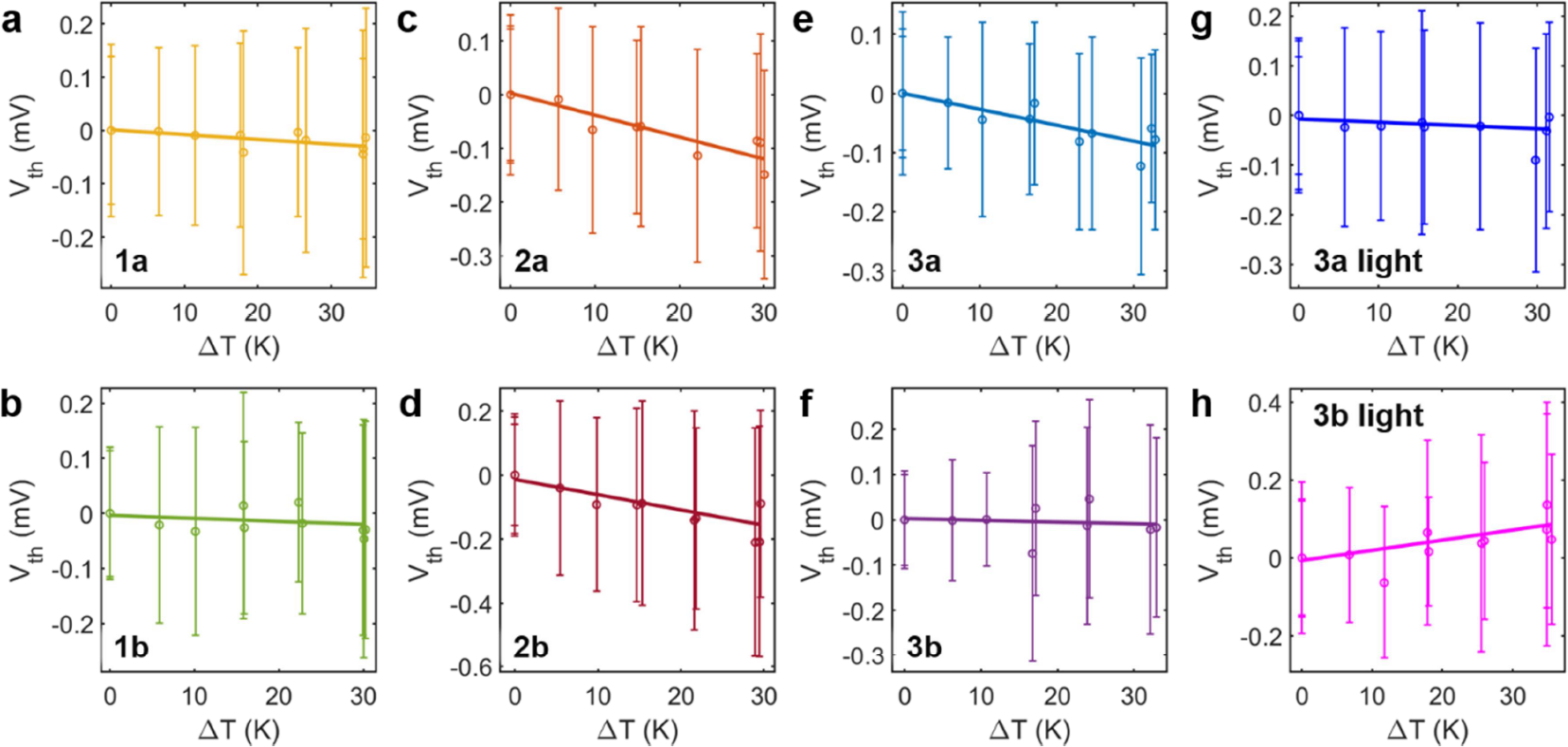
Most probable thermovoltage values with the dispersion as error bars and linear regressions performed to all data points of the temperature difference dependence of the thermovoltage measurements of compounds (a) **1a**, (b) **1b**, (c) **2a**, (d) **2b**, (e) **3a**, and (f) **3b** in the dark and (g) **3a** and (h) **3b** in light, in the selected traces that show a molecular response measured for several temperature differences. The measurements in light are without the high Seebeck value data points.

The Seebeck coefficient is determined by performing current vs voltage ramps of ±10 mV when a plateau is detected.^41^ A 1 kΩ resistance implemented in the tip holder is used to heat the tip, creating a temperature difference between the electrodes (tip–sample). The thermoelectric effect causes a displacement of the *I*–*V* curves. The offsets in the current and voltage of the system are calibrated after every junction by measuring the *I*–*V* curves in the open circuit and switching the electronic path to a resistance in parallel to the STM. The remaining offset corresponds to the thermovoltage, while the slope of the *I*–*V* curves is the conductance, thus allowing the measurement of both parameters simultaneously. The Seebeck coefficient is obtained as the slope in the fitting of the linear regression of the temperature difference dependence of the thermovoltage, as shown in Figure 4.

In Figure 3a,b, the conductance histograms of each compound display a main conductance distribution around the most probable conductance value. The relatively narrow breadth of the conductance histogram peaks is indicative of a rigid molecule anchored with thiomethyl groups. The relatively small plateaus compared to the length of the compounds (see the SI, Section B) indicate that the molecule is not fully spanning the junction, possibly remaining in a tilted position before detachment.^42^ This makes the quantitative determination of the breakoff distance difficult to achieve. However, a comparison of conductance vs distance histograms (Figure S28) shows that qualitatively the longer molecules (**1b**, **2b**, and **3b**) have a greater junction elongation than the shorter analogues (**1a**, **2a**, and **3a**), consistent with an increase in molecular length in the “**b**” series.

As often observed in single-molecule measurements,^43^ the short compounds (**1a**, **2a**, and **3a**) demonstrated higher conductance values than their longer analogues (**1b**, **2b**, and **3b**); i.e., **1a** and **1b** have conductance values of −4.4 and −5.0 log(*G*/*G*_0_), and **2a** and **2b**, −5.2 and −5.6 log(*G*/*G*_0_), respectively. Compounds **3a** and **3b** showed the greatest decrease in conductance upon elongation of the molecular backbone, with values of −3.8 to −5.5 log(*G*/*G*_0_) respectively, which can be explained by this pair having the largest increase in conjugation length. Based on oligo(phenylene ethynylene) β-values of 3.4 nm^–1^,^44^ it is possible to empirically compare the relative conductance of the long and short molecules to show that despite the length of the molecules, the conductance attenuation is consistent with a tunneling regime (see the SI, Section B).

**Figure 5:**
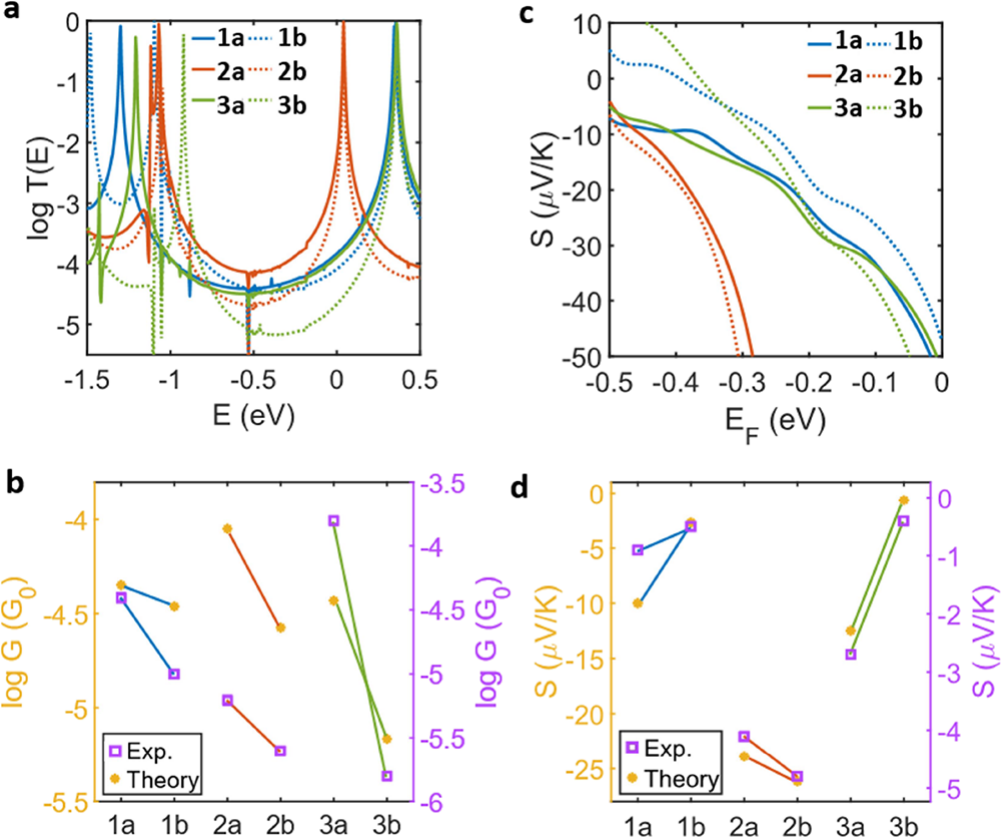
Transmission, conductance, and Seebeck coefficients of compounds **1a**, **1b**, **2a**, **2b**, **3a**, and **3b**. (a) DFT electron transmission probabilities of compounds **1a**, **1b**, **2a**, **2b**, **3a**, and **3b**. (b) Measured and theoretical conductances at a Fermi energy of −0.36 eV. (c) Theoretical Seebeck coefficients. (d) Measured and theoretical Seebeck coefficients at a Fermi energy of −0.36 eV.

The Seebeck coefficients in each case are negative (Figure 4), indicating LUMO-dominated transport, which is supported by our calculations below (Figure 5). Previous studies with SMe anchors provide precedents for either HOMO- or LUMO-dominated transport.^36,45−48^ Compounds **1a** and **1b** had similarly low values of −0.9 and −0.5 μV/K, respectively (Figure 4a,b), indicating that the position of *E*_F_ is close to the center of the H-L gap. Compounds **2a** and **2b** (Figure 4c,d) had higher values, −4.1 and −4.8 μV/K, respectively, even though these compounds had a higher H-L gap (Table S2), suggesting a higher transmission gradient around the Fermi energy. Compounds **3a** and **3b** (Figure 4e,f) have Seebeck coefficients of −2.7 and −0.4 μV/K, respectively, showing the atypical trend of the Seebeck coefficient reducing as the conjugation length increases.^19^

### Theoretical Method and Results

First principles calculations were performed to further understand the thermoelectric properties of compounds **1a**, **1b**, **2a**, **2b**, **3a**, and **3b**. Initially, the ground-state geometry of the molecular structures was obtained using a self-consistent scheme implemented in the SIESTA^49^ density functional theory code. Once the relaxed structure was determined, it was positioned in a junction between two gold leads to obtain the ground-state structures after geometry optimization. The DFT mean-field Hamiltonians of these structures were then combined with GOLLUM^50^ using the nonequilibrium Green’s function method to calculate the electron transmission,^51^ with further details provided in Section D of the SI. The eigenvalues show that both BDP compounds, **2a** and **2b**, have the smallest H-L gap (1.09 and 0.99 eV, respectively), with both their LUMO energies closest to the *E*_F_ compared to those of the other compounds, with an about 0.3 eV difference. All compounds show clear continuum-state orbitals with the LUMO orbitals localized around the core of the backbone of the molecule (Figure S37a–c).

The electron transmission was calculated for IIG (**1a** and **1b**), BDP (**2a** and **2b**), and BPS (**3a** and **3b**) compounds. The transmission curves (see Figure 5a) for each of the compounds are featureless within the H-L gap; i.e., there is no suggestion of DQI features. Compounds **1b** (1.46 eV) and **3b** (1.28 eV) clearly show smaller H-L gaps compared to those of their shorter analogues **1a** (1.65 eV) and **3a** (1.57 eV). However, **2a** (1.11 eV) and **2b** (1.09 eV) have a very similar H-L gap size consistent with measured H-L gap data (see Table S2). Given how significantly the alignment of *E*_F_ can determine the conductive behavior of a system, we started by comparing the measured conductance and Seebeck coefficients. Due to the negative sign of the Seebeck coefficient, the conductance is LUMO dominated and the comparison of the calculated transmission curve and measured values determined that an *E*_F_ = −0.36 eV gives the closest match; i.e., this predicts conductances of −4.3, −4.5, −4.0, −4.6, −4.4, and −5.2 log(*G*/*G*_0_) for **1a**, **1b**, **2a**, **2b**, **3a**, and **3b**, respectively, reproducing the attenuation attributed to molecular length (Figure 5b). The full conductance plots are shown in Figure S38.

The Seebeck coefficients of these compounds were derived from the DFT transmission using the method explained in the SI, Section D (Figure 5c), giving values of −10, −2.7, −24, −26, −12.5, and −0.6 μV/K for **1a**, **1b**, **2a**, **2b**, **3a**, and **3b**, respectively (Figure 5d). Compounds **1a** and **3a** have higher Seebeck coefficients compared to their longer analogues **1b** and **3b**, due to the pinning of the LUMO resonance (as a result of the type of anchor), meaning that the gradient of the transmission around the Fermi energy is less for **1b** and **3b**. While compounds **2a** and **2b** have very similar H-L gap sizes, the higher Seebeck coefficient for **2b** is due to a narrower LUMO transport resonance, leading to a higher slope at the Fermi energy and consequently a higher *S*.

These results highlight that rather than it being exclusively the result of the H-L gap or a particular class of compounds, it is the position of the frontier orbitals relative to *E*_F_ that has the dominant role in determining the Seebeck coefficients of the molecular wires. This is particularly exemplified by the BDP compounds (**2a** and **2b**). We expanded on this observation by using theoretical models to examine how varying the anchor group changes the Seebeck coefficients by replacing the thioanisole groups of **2a**, **2b**, **3a**, and **3b** with either pyridyl (**2a-Py**, **2b-Py**, **3a-Py**, and **3b-Py**) or benzonitrile (**2a-CN** and **2b-CN**) anchors (see Figure S39). Using this approach, it has been shown that for **3a** and **3b**, a shift of the LUMO relative to *E*_F_ by approximately 0.1 eV (Py) and 0.08 eV (CN), although small, would give a theoretical Seebeck coefficient increase due to an increased transmission slope (Figure S40). Furthermore, to understand the effect of molecular conformation on the thermoelectric properties, we studied these properties, in bent junctions formed by **3a** and **3b**. We found that while, in agreement with previous reports,^52−55^ the values obtained are sensitive to the conformations, the trend observed is not and is in agreement with the trend observed experimentally as shown in Figure S41.

It is worth noting that the thermoelectric measurements of the BPS compounds (**3a** and **3b**) proved to be fickler than those of the other molecules due to their light sensitivity when on a gold surface (see the SI, Section B). All compounds were characterized under ambient light conditions. In the case of the two compounds (**3a** and **3b**), their most probable Seebeck values were −0.7 μV/K for **3a** and +2.6 μV/K for **3b** (Figure 4e,f). It was observed that these values drifted over time, becoming more positive. Repeating the measurements without light gave the original, stable values (−2.7 μV/K for **3a** and −0.4 μV/K for **3b**), confirming the light’s effect on the Seebeck coefficient (see the SI for more details). This suggests that the molecules degraded in the presence of light, which was further confirmed by X-ray photoelectron spectroscopic (XPS) data in the SI. However, this degradation did not have a significant effect on their electrical conductance.

To understand this further, we investigated theoretically the possible scenarios (as shown in the SI, Section D). We found that changes to the molecules’ structure, or the formation of bimolecules bonded together either through π–π stacking or through a covalent bond, lead to changes in the QI pattern through molecules **3a** and **3b**, which in turn influence the slope of transmission curves (and consequently *S*) more significantly than their amplitude (and consequently *G*). This is also in line with the additional features we observed experimentally under light, depicted in Figure S31e,f, where we found two distinct values for both compounds: around −7 and −23 μV/K for **3a** and around −75 and −117 μV/K for **3b** as shown in Figure 4g,h (see the SI, Section B, for more details), each with a yield lower than 5%.

## Conclusions

A series of aromatic (IIG), quinoidal (BDP), and D-A (BPS) small-H-L-gap molecules end-capped with thioanisole groups was synthesized to examine the impact of the H-L gap on the Seebeck coefficients in gold|single molecule|gold junctions. Due to their combination of relatively simple structures and linear π-conjugated conductance paths, comparatively high electrical conductances were established and impacts of destructive QI features were avoided. Through the measurement of Seebeck coefficients, it was established that the conductance of all of the compounds was LUMO dominated. However, despite the relatively low H-L gap of the compounds, the magnitude of the Seebeck coefficients remained low, which can be explained by the relative positions of *E*_F_ to the frontier orbitals. Therefore, from this study, it can be concluded that to fully exploit enhancement in thermoelectric behavior resulting from H-L gap reduction, it is necessary to make a judicious choice of molecular components to better match the orbitals’ alignment with *E*_F_. As a first step in future studies, different anchor groups could be investigated.

## Methodology

### Synthesis

The SI contains the synthesis procedures and characterizations.

### Measurement

Conductance and Seebeck coefficient measurements were carried out using a homebuilt STM at room temperature and under ambient conditions, and a moderate temperature range was chosen as our heating method is limited by the voltage that can be applied to the resistance in the tip holder (see the SI for details).

### Theory

The optimized geometries with ground-state Hamiltonian and overlap matrix elements for gas-phase molecules and molecules between electrodes were obtained using DFT. These results were then combined with the Green’s function method to calculate the phase-coherent, elastic-scattering properties of the system, consisting of two gold electrodes and the molecule as the scattering region. From the calculated transmission functions, the electrical conductance and the Seebeck coefficient were calculated. See the SI for details of computational methods.

## Data Availability

The experimental data underlying this study are openly available in ZENODO at DOI: 10.5281/zenodo.10549417.

## Supporting Information Available

Experimental procedures, synthesis, NMR spectra, crystallographic and photophysical data, compound stability, conductance and Seebeck measurements, XPS data and theoretical data, crystallographic data for compounds **1a** and **1b** (CSD 2301298 and 2301299, respectively) (PDF)
