# Supplementary material for: Exploring the Impact of the HOMO–LUMO Gap on Molecular Thermoelectric Properties: A Comparative Study of Conjugated Aromatic, Quinoidal, and Donor–Acceptor Core Systems
Source: ACS Omega. 2024 Feb 5;9(7):8471–7. doi: 10.1021/acsomega.3c09760 (PMC10882689; doi:10.1021/acsomega.3c09760)
Supplement: Supplementary file 1 — ao3c09760_si_001.pdf [file ao3c09760_si_001.pdf]

Supplementary Information

Exploring the Impact of HOMO-LUMO Gap on Molecular Thermoelectric Properties: A Comparative Study of Conjugated Aromatic, Quinoidal, and Donor-Acceptor Core Systems

Nickel Blankevoort,[1] Pablo Bastante,[2] Ross J. Davidson,[3] Rebecca J. Salthouse,[3] Abdalghani H. S. Daaoub,[1] Pilar Cea,[4,5] Santiago Martin,[4,5] Andrei S. Batsanov,[3] Sara Sangtarash,[1] Martin R. Bryce,[3,*] Nicolas Agrait,[2,6,*] and Hatef Sadeghi[1,*]

[1] Device Modelling Group, School of Engineering, University of Warwick, Coventry CV4 7AL, UK

[2] Departamento de Física de la Materia Condensada C-III, Universidad Autónoma de Madrid, E-28049 Madrid, Spain

[3] Department of Chemistry, Durham University, Durham, DH1 3LE, UK

[4] Instituto de Nanociencia y Materiales de Aragón (INMA), CSIC-Universidad de Zaragoza, 50009, Zaragoza, Spain.

[5] Departamento de Química Física, Universidad de Zaragoza, 50009, Zaragoza, Spain and Laboratorio de Microscopias Avanzadas (LMA), Universidad de Zaragoza, 50018, Zaragoza, Spain.

[6] Condensed Matter Physics Center (IFIMAC), and Instituto Universitario de Ciencia de Materiales "Nicolás Cabrera", Universidad Autónoma de Madrid, 28049 Madrid, Spain.

Table of Contents

A) Experimental

S1. Synthesis S3

S2. NMR spectra of reported compounds S15

S3. X-ray Crystallography S26

S4. Photophysical Measurements S29

S5. Stability of bis(pyrrol-2-yl)squaraines **3a** and **3b** S30

B) Conductance and Seebeck measurements S34

C) XPS measurements S39

D) Theoretical calculations S41

References S54

a) Experimental

Instrumentation. NMR spectra were recorded in deuterated solvent solutions on a Varian VNMRS-600 spectrometer and referenced against solvent resonances (^{1}H, ^{13}C). Accurate mass tandem mass spectrometer equipped with Atmospheric Pressure Gas Chromatography (APGC) and Atmospheric Solids Analysis Probe (ASAP) data were recorded on a high-resolution Xevo QTOF instrument (Waters). Microanalyses were performed by the Elemental Analysis Service at Durham University, UK.

General details. The compounds 6,6'-dibromo-1,1'-dihexyl-[3,3'-biindolinylidene]-2,2'-dione [1], 1-hexyl-2-(4,4,5,5-tetramethyl-1,3,2-dioxaborolan-2-yl)-1H-pyrrole [2], tributyl(4-(methylthio)phenyl)stannane [3], and (4-ethynylphenyl)(methyl)sulfane [4] were prepared according to published methods; all other chemicals were sourced from standard chemical suppliers. For information on atom labels see NMR spectra of reported compounds.

1,1'-dihexyl-6,6'-bis(4-(methylthio)phenyl)-[3,3'-biindolinylidene]-2,2'-dione **1a**. A solution containing 6,6'-dibromo-1,1'-dihexyl-[3,3'-biindolinylidene]-2,2'-dione (0.50 g, 0.85 mmol) and tributyl(4-(methylthio)phenyl)stannane (0.70 g, 1.70 mmol) in toluene (50 mL) was degassed by bubbling argon through it after which $Pd(PPh_3)_4$ (98 mg, 0.085 mmol) was added. The solution was heated to reflux for 16 hours after which the solution was cooled and the solvent removed *in vacuo*. The residue was purified by silica chromatography eluted by a solvent gradient from neat hexane to neat DCM, to give a red solid. **Yield**: 0.44 g (78%). **^{1}H NMR** (600 MHz; CD_2Cl_2): δ_H 9.24 (d, $^{3}J_{HH}$ = 8.3 Hz, 2H, H$_c$), 7.62 (d, $^{3}J_{HH}$ = 8.2 Hz, 4H, H$_d$), 7.35 (d, $^{3}J_{HH}$ = 8.1 Hz, 4H, H$_e$), 7.26 (dd, $^{3}J_{HH}$ = 8.3 Hz, $^{4}J_{HH}$ = 1.7 Hz, 2H, H$_b$), 7.01 (d, $^{4}J_{HH}$ = 1.7 Hz, 2H, H$_a$), 3.83 (t, $^{3}J_{HH}$ = 7.4 Hz, 4H, H$_g$), 2.54 (s, 6H, H$_f$), 1.74 (p, $^{3}J_{HH}$ = 7.5 Hz, 4H),

1.46-1.41 (m, 4H, H_i), 1.38-1.31 (m, 8H, H_j+H_k), 0.90 (t, $^3J_{HH}$ = 6.9 Hz, 6H, H_l) ppm. **^{13}C{^{1}H} NMR** (150 MHz; CD_2Cl_2): δ_C 168.1, 145.4, 144.0, 139.2, 136.8, 132.2, 130.1, 127.2, 126.4, 120.8, 119.9, 105.8, 39.9, 31.4, 27.4, 26.6, 22.5, 15.3, 13.7 ppm. **Acc-MS**(ASAP$^+$): *m/z* 675.3068 $[M+H]^+$ calcd. for $C_{42}H_{47}N_2O_2S_2$ *m/z* 675.3079 ($|\Delta m/z|$ = 1.6 ppm). **Anal. Calc.** for $C_{42}H_{46}N_2O_2S_2$: C, 74.74; H, 6.87; N, 4.15 %. **Found**: C, 74.49; H, 6.84; N, 3.99 %.

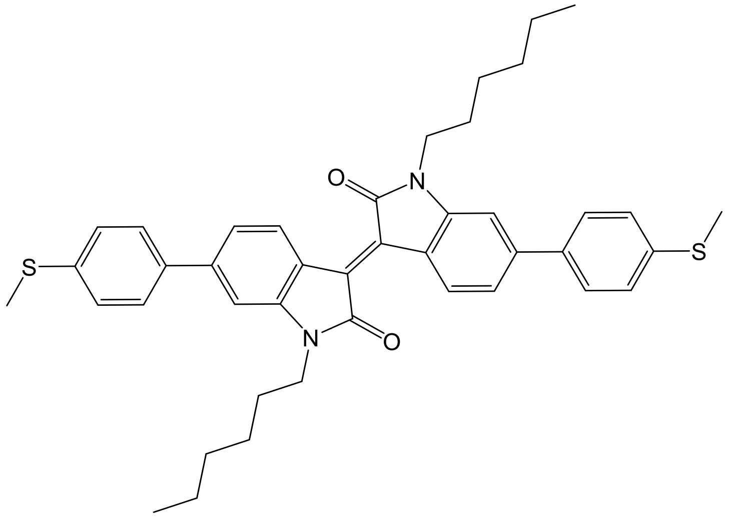

3,7-bis(4-bromophenyl)-1,5-dihexylpyrrolo[2,3]indole-2,6-dione. A solution containing 3,7-bis(4-bromophenyl)pyrrolo[2,3-f]indole-2,6(1H,5H)-dione (2.00 g, 4.03 mmol), K_2CO_3 (1.25 g, 9.00 mmol), and 1-bromohexane (2.52 mL, 3.00 g, 18.00 mmol) in DMF (30 mL) was heated to 100°C for 16 hours. The solution was cooled to room temperature and extracted with water and diethyl ether, collecting the organic layer and drying it over $MgSO_4$. The solvent was removed *in vacuo*, the residue was purified by silica chromatography eluted by a solvent gradient from DCM:Hexane (1:9) to ethylacetate:DCM (1:9). **Yield**: 3.00 g (50%). **^{1}H NMR** (600 MHz; CD_2Cl_2): δ_H 7.67 (d, $^3J_{HH}$ = 8.0 Hz, 4H, H_c), 7.62 (d, $^3J_{HH}$ = 8.0 Hz, 4H, H_b), 6.33 (s, 2H, H_a), 3.65 (t, $^3J_{HH}$ = 7.2 Hz, 4H, H_d), 1.66 (p, $^3J_{HH}$ = 7.2 Hz, 4H, H_e), 1.40-1.32 (m, 12H, H_f+H_g+H_h), 0.92 (t, $^3J_{HH}$ = 7.2 Hz, 6H, H_j) ppm. **^{13}C{^{1}H} NMR** (150 MHz; CD_2Cl_2): δ_C 169.1, 143.9, 134.7, 131.8, 131.0, 130.2, 126.2, 123.1, 96.7, 39.4, 31.3, 28.1, 26.4, 22.5, 13.7 ppm.

Acc-MS(ASAP$^+$): *m/z* 663.1222 [M+H]$^+$ calcd. for $C_{34}H_{37}N_2O_2Br_2$ *m/z* 663.1222 (|Δ*m/z*| = 0.3 ppm). **Anal. Calc.** for $C_{34}H_{36}Br_2N_2O_2 \cdot \frac{3}{4}H_2O$: C, 60.23; H, 5.57; N, 4.13 %. **Found**: C, 60.30; H, 5.40; N, 4.07 %.

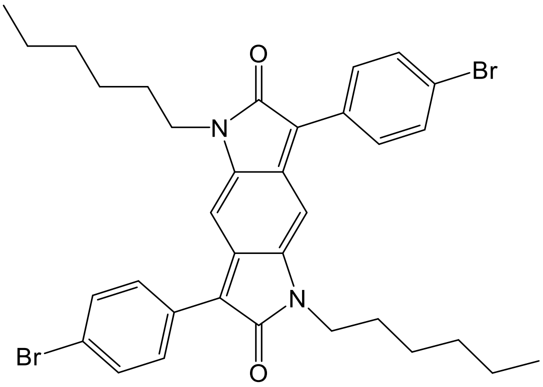

1,1'-dihexyl-6,6'-bis(4-(methylthio)phenyl)-[3,3'-biindolinylidene]-2,2'-dione **2a**. A solution containing 3,7-bis(4-bromophenyl)-1,5-dihexylpyrrolo[2,3-f]indole-2,6(1H,5H)-dione (0.50 g, 0.75 mmol) and tributyl(4-(methylthio)phenyl)stannane (0.63 g, 1.51 mmol) in toluene (50 mL) was degassed by bubbling argon through it after which $Pd(PPh_3)_4$ (86 mg, 0.075 mmol) was added. The solution was heated to reflux for 16 hours after which the solution was cooled and the solvent removed *in vacuo*. The residue was purified by silica chromatography eluted by a solvent gradient from DCM:Hexane (1:9) to ethylacetate:DCM (1:9), to give a red solid. **Yield**: 0.28 g (51%). **^{1}H NMR** (600 MHz; CD_2Cl_2): δ_H 7.81 (d, $^3J_{HH}$ = 8.4 Hz, 4H, H_c), 7.74 (d, $^3J_{HH}$ = 8.4 Hz, 4H, H_b), 7.63 (d, $^3J_{HH}$ = 8.4 Hz, 4H, H_d), 7.36 (d, $^3J_{HH}$ = 8.4 Hz, 4H, H_e), 6.43 (s, 2H, H_a), 3.66 (t, $^3J_{HH}$ = 7.3 Hz, 4H, H_g), 2.54 (s, 6H, H_f), 1.67 (p, $^3J_{HH}$ = 7.3 Hz, 4H, H_h), 1.39-1.32 (m, 12H, H_i+H_j+H_k), 0.89 (t, $^3J_{HH}$ = 6.9 Hz, 6H, H_l) ppm. **^{13}C{^{1}H} NMR** (150 MHz; CD_2Cl_2): δ_C 169.4, 143.7, 140.6, 138.6, 136.6, 134.3, 130.0, 127.2, 126.8, 126.7, 126.6, 97.0, 39.4, 31.4, 28.1, 26.5, 22.5, 15.3, 13.7 ppm. **Acc-MS**(ASAP$^+$): *m/z* 751.3408 [M+H]$^+$ calcd. for $C_{48}H_{51}N_2O_2S_2$ *m/z* 751.3392 (|Δ*m/z*| = 2.1 ppm). **Anal. Calc.** for $C_{48}H_{50}N_2O_2S_2$: C, 76.76; H, 6.71; N, 3.73 %. **Found**: C, 76.25; H, 6.62; N, 3.65 %.

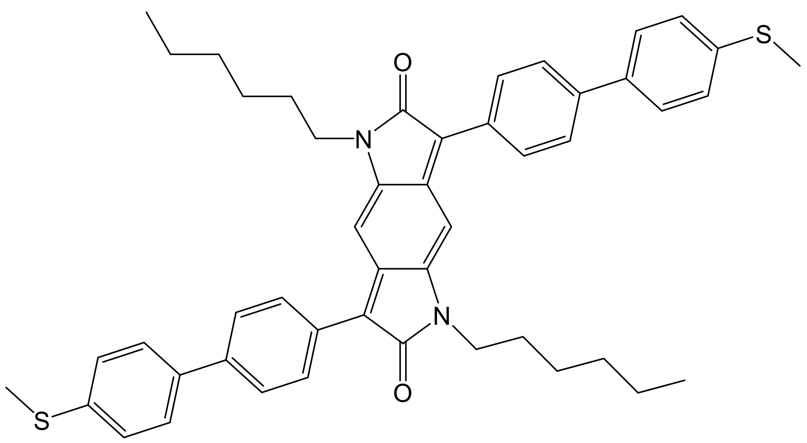

1-hexyl-2-(4-(methylthio)phenyl)-1H-pyrrole. A solution containing 1-hexyl-2-(4,4,5,5-tetramethyl-1,3,2-dioxaborolan-2-yl)-1H-pyrrole (2.00 g, 10.21 mmol), (4-bromophenyl)(methyl)sulfane (2.82 g, 10.21 mmol), K_2CO_3 (2.40 g, 17.38 mmol) in toluene (50 mL), EtOH (10 mL), and water (10 mL) was degassed by bubbling argon through it after which $Pd(PPh_3)_4$ (500 mg, 0.43 mmol) was added. The solution was heated to reflux for 16 hours after which the solution was cooled and the solvent removed *in vacuo*. The residue was purified by silica chromatography eluted by a solvent gradient from neat hexane to neat DCM. **Yield**: 0.60 g (21 %). **^{1}H NMR** (600 MHz; CD_2Cl_2): δ_H 7.35-7.30 (m, 4H, H_d+H_e), 6.80 (dd, $^3J_{HH}$ = 2.8 Hz, $^4J_{HH}$ = 1.8 Hz, 1H, H_a), 6.19 (dd, $^3J_{HH}$ = 3.6 Hz, $^3J_{HH}$ = 2.7 Hz, 1H, H_c), 6.16 (dd, $^3J_{HH}$ = 3.5 Hz, $^4J_{HH}$ = 1.8 Hz, 1H, H_b), 3.95 (t, $^3J_{HH}$ = 4.7 Hz, 2H, H_g), 2.55 (s, 3H, H_f), 1.70-1.63 (m, 2H, H_h), 1.31-1.20 (m, 6H, H_i+H_j+H_k), 0.88 (t, $^3J_{HH}$ = 6.9 Hz, 3H, H_l) ppm. **^{13}C{^{1}H} NMR** (150 MHz; CD_2Cl_2): δ_C 137.0, 133.5, 130.5, 129.1, 126.2, 122.0, 108.4, 107.6, 47.1, 31.5, 31.2, 26.2, 22.5, 15.5, 13.7 ppm. **Acc-MS**(ASAP$^+$): *m/z* 274.1651 $[M+H]^+$ calcd. for $C_{17}H_{24}NS$ *m/z* 274.1629 ($|\Delta m/z|$ = 8.0 ppm).[i]

i Too unstable for elemental analysis.

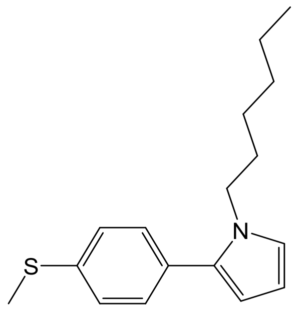

2-(1-hexyl-5-(4-(methylthio)phenyl)-1H-pyrrol-2-yl)-4-(1-hexyl-5-(4-(methylthio)phenyl)-2H-pyrrol-1-ium-2-ylidene)-3-oxocyclobut-1-en-1-olate **3a**. A mixture of 1-hexyl-2-(4-(methylthio)phenyl)-1H-pyrrole (0.50 g, 1.83 mmol), and squaric acid (0.103 g, 0.91 mmol) in n-BuOH/Toluene (10 ml:10 ml) was refluxed for 6 h. After removed of the solvent and extracted with DCM. The organic phase was washed with water, dried over $MgSO_4$ and filtered. The solvent was removed from the filtrate under reduced pressure to give blue solid. The product was purified by column chromatography (silica gel, hexane/methylene chloride 1:9) to give blue solid. **Yield**: 0.18 g (33%). **^{1}H NMR** (600 MHz; CD_2Cl_2): δ_H 7.89 (d, $^3J_{HH}$ = 4.3 Hz, 2H, H_d), 7.44 (d, $^3J_{HH}$ = 8.0 Hz, 4H, H_b), 7.36 (d, $^3J_{HH}$ = 8.0 Hz, 4H, H_a), 6.58 (d, $^3J_{HH}$ = 4.3 Hz, 2H, H_c), 4.82 (t, $^3J_{HH}$ = 7.4 Hz, 4H, H_f), 2.55 (s, 6H, H_e), 1.59-1.54 (m, 4H, H_g), 1.18-1.08 (m, 12H, H_i+H_j+H_k), 0.79 (t, $^3J_{HH}$ = 0.79 Hz, 6H, H_k) ppm. **^{13}C{^{1}H} NMR** (150 MHz; CD_2Cl_2): δ_C 174.2, 149.2, 140.9, 130.1, 128.9, 127.4, 125.9, 123.9, 116.7, 47.4, 31.9, 31.2, 25.8, 22.3, 14.9, 13.6 ppm. **Acc-MS**(ASAP$^+$): *m/z* 625.2915 $[M+H]^+$ calcd. for $C_{38}H_{45}N_2O_2S_2$ *m/z* 625.2922 ($|\Delta m/z|$ = 1.1 ppm). **Anal. Calc.** for $C_{38}H_{44}N_2O2S_2{\cdot}H_2O$: C, 72.00; H, 7.15; N, 4.42 %. **Found**: C, 71.97; H, 6.99; N, 4.30 %.

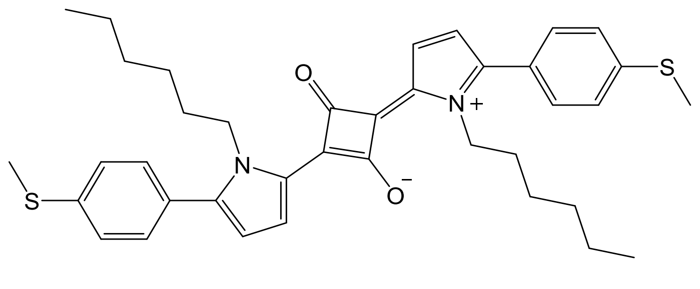

1,1'-dihexyl-6,6'-bis((4-(methylthio)phenyl)ethynyl)-[3,3'-biindolinylidene]-2,2'-dione **1b**. A solution containing 1,1'-dihexyl-6,6'-bis(4-(methylthio)phenyl)-[3,3'-biindolinylidene]-2,2'-dione (0.50 g, 0.85 mmol), (4-ethynylphenyl)(methyl)sulfane (0.264 g, 1.78 mmol), triethylamine (Et_3N, 5.0 mL), THF (30 mL) was degassed by three freeze-pump-thaw cycles, after which $PdCl_2(PPh_3)_2$ (63 mg, 0.09 mmol) and CuI (17 mg, 0.09 mmol) were added. The solution was heated to reflux for 16 hours before the solvent was removed in *vacuo*. The residue was purified by silica chromatography eluted by a solvent gradient from neat hexane to hexane:DCM (1:1). **Yield:** 0.29 g (48%). **^{1}H NMR** (600 MHz; CD_2Cl_2): δ_H 9.18 (dd, $^3J_{HH}$ = 8.3 Hz, $^4J_{HH}$ = 2.2 Hz, 2H, H_c), 7.48 (d, $^3J_{HH}$ = 8.4 Hz, 4H, H_e), 7.24 (d, $^3J_{HH}$ = 8.4 Hz, 4H, H_d), 7.18 (dd, $^3J_{HH}$ = 8.3 Hz, $^4J_{HH}$ = 2.5 Hz, 2H, H_b), 6.95 (d, $^3J_{HH}$ = 2.0 Hz, 2H, H_a), 3.78 (t, $^3J_{HH}$ = 8.1 Hz, 4H, H_g), 2.52 (s, 6H, H_f), 1.73-1.70 (m, 4H, H_h), 1.43-1.33 (m, 12H, H_i+H_j+H_k), 0.90 (t, $^3J_{HH}$ = 6.0 Hz, 6H, H_l) ppm. **^{13}C{^{1}H} NMR** (150 MHz; CD_2Cl_2): δ_C 167.6, 144.8, 140.4, 132.5, 131.9, 129.6, 126.8, 125.6, 125.0, 121.6, 118.7, 110.3, 92.3, 90.0, 40.0, 31.4, 27.3, 26.6, 22.5, 14.9, 13.7 ppm. **Acc-MS**(ASAP$^+$): *m/z* 723.3072 $[M+H]^+$ calcd. for $C_{46}H_{47}N_2O_2S_2$ *m/z* 723.3079 ($|\Delta m/z|$ = 1.0 ppm). **Anal. Calc.** for $C_{46}H_{46}N_2O_2S_2\cdot\frac{1}{4}CH_2Cl_2$: C, 74.64; H, 6.30; N, 3.76 %. **Found**: C, 74.64; H, 6.11; N, 3.67 %. Crystals for X-ray analysis were grown by slow evaporation of a DCM solution.

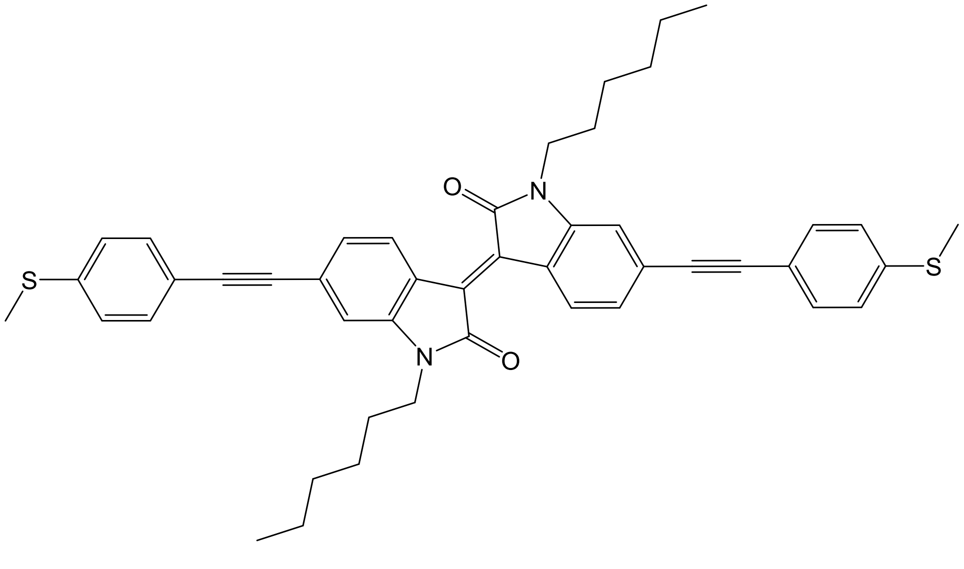

1,5-dihexyl-3,7-bis(4-((4-(methylthio)phenyl)ethynyl)phenyl)pyrrolo[2,3-f]indole-2,6(1H,5H)-dione **2b**. A solution containing 3,7-bis(4-bromophenyl)-1,5-dihexylpyrrolo[2,3-f]indole-2,6-dione (0.50 g, 0.75 mmol), (4-ethynylphenyl)(methyl)sulfane (0.237 g, 1.60 mmol), triethylamine (Et_3N, 5.0 mL), THF (30 mL) was degassed by three freeze-pump-thaw cycles, after which $PdCl_2(PPh_3)_2$ (49 mg, 0.07 mmol) and CuI (10 mg, 0.07 mmol) were added. The solution was heated to reflux for 16 hours before the solvent was removed *in vacuo*. The residue was purified by silica chromatography eluted by a solvent gradient from hexane:DCM (1:1) to DCM:EtOAc (9:1). **Yield**: 0.23 g (40%). **^{1}H NMR** (600 MHz; CD_2Cl_2): δ_H 7.74 (d, $^3J_{HH}$ = 8.4 Hz, 4H, H_c), 7.63 (d, $^3J_{HH}$ = 8.4 Hz, 4H, H_b), 7.48 (d, $^3J_{HH}$ = 8.4 Hz, 2H, H_e), 7.24 (d, $^3J_{HH}$ = 8.4 Hz, 2H, H_d), 6.38 (s, 2H, H_a), 3.64 (t, $^3J_{HH}$ = 7.3 Hz, 4H, H_g), 2.52 (s, 6H, H_f), 1.65 (q, $^3J_{HH}$ = 7.3 Hz, 4H, H_h), 1.39-1.32 (m, 12H, H_i+H_j+H_k), 0.90 (t, $^3J_{HH}$ = 4.5 Hz, 6H, H_l) ppm. **^{13}C{^{1}H} NMR** (150 MHz; CD_2Cl_2): δ_C 169.2, 143.9, 140.1, 134.6, 131.8, 131.6, 131.2, 129.4, 126.4, 125.6, 123.7, 119.0, 97.0, 77.2, 39.4, 31.4, 30.5, 28.1, 26.5, 22.5, 14.9, 13.7 ppm.[ii] **Acc-MS**(ASAP$^+$): *m/z* 799.3393 $[M+H]^+$ calcd. for $C_{52}H_{51}N_2O_2S_2$ *m/z* 799.3392 ($|\Delta m/z|$ = 0.1 ppm). **Anal. Calc.** for $C_{52}H_{50}N_2O_2S_2 \cdot ⅓CH_2Cl_2$: C, 76.01; H, 6.17; N, 3.39 %. **Found**: C, 76.01; H, 5.88; N, 3.44 %. Crystals for X-ray analysis were grown by slow evaporation of a DCM solution.

ii Low solubility resulted in poor signal to noise ratio.

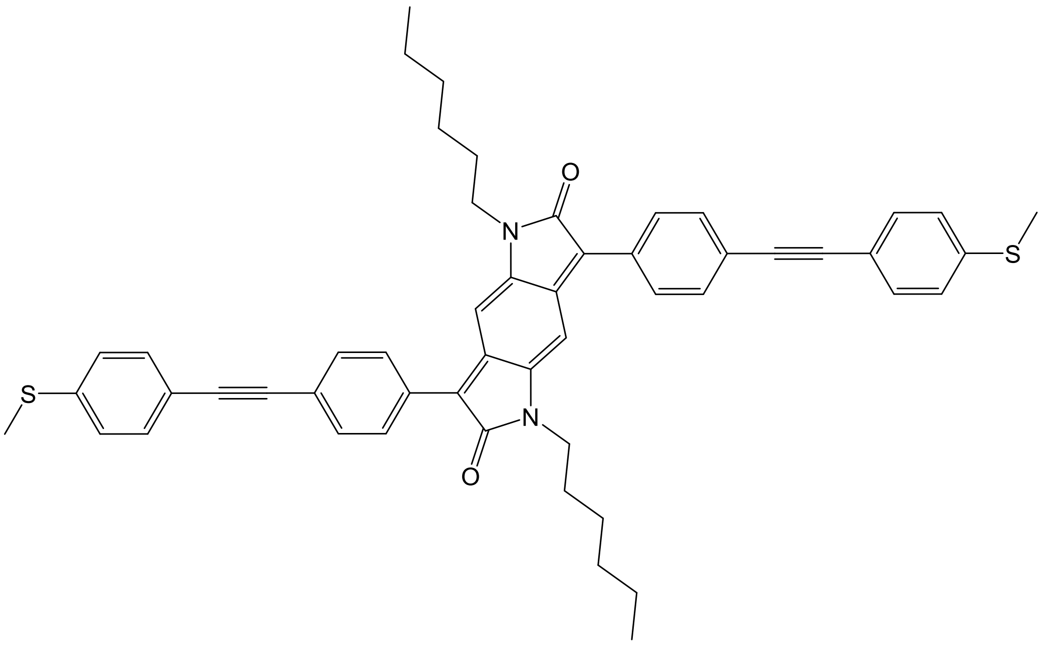

tert-butyl 2-(4-iodophenyl)-1H-pyrrole-1-carboxylate. A solution containing (1-(tert-butoxycarbonyl)-1H-pyrrol-2-yl)boronic acid (1.0 g, 4.73 mmol), 1,4-diiodobenzene (3.11 g, 9.47 mmol), K_2CO_3 (1.30 g, 9.47 mmol) in toluene (50 mL), EtOH (10 mL), and water (10 mL) was degassed by bubbling argon through it after which $Pd(PPh_3)_4$ (542 mg, 0.47 mmol) was added. The solution was heated to reflux for 16 hours after which the solution was cooled and the solvent removed *in vacuo*. The residue was purified by silica chromatography eluted by a solvent gradient from neat hexane to neat DCM. **Yield**: 0.61 g (35%). **^{1}H NMR** (600 MHz; CD_2Cl_2): δ_H 7.73 (d, $^3J_{HH}$ = 8.6 Hz, 2H, H_e), 7.39 (dd, $^3J_{HH}$ = 3.3 Hz, $^4J_{HH}$ = 1.8 Hz, 1H, H_a), 7.15 (d, $^3J_{HH}$ = 8.6 Hz, 2H, H_d), 6.27-6.23 (m, 2H, H_b+H_c), 1.44 (s, 9H, H_f) ppm. **^{13}C{^{1}H} NMR** (150 MHz; CD_2Cl_2): δ_C. 149.0, 136.6, 134.0, 133.8, 131.0, 122.9, 114.6, 110.5, 92.4, 83.9, 27.4 ppm. **Acc-MS**(ASAP$^+$): *m/z* 370.0320 [M+H]$^+$ calcd. for $C_{15}H_{17}NO_2{}^{127}I$ *m/z* 370.0304 (|Δ*m/z*| = 4.3 ppm). **Anal. Calc.** for $C_{15}H_{16}INO_2 \cdot ⅓H_2O$: C, 48.03; H, 4.48; N, 3.73 %. **Found**: C, 48.03; H, 4.29; N, 3.37 %.

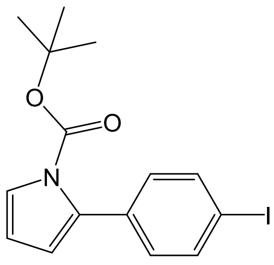

tert-butyl 2-(4-((4-(methylthio)phenyl)ethynyl)phenyl)-1H-pyrrole-1-carboxylate. A solution containing *tert-butyl 2-(4-iodophenyl)-1H-pyrrole-1-carboxylate* (1.00 g, 2.71 mmol), (4-ethynylphenyl)(methyl)sulfane (0.401 g, 2.71 mmol), triethylamine (Et_3N, 5.0 mL), THF (30 mL) was degassed by three freeze-pump-thaw cycles, after which $PdCl_2(PPh_3)_2$ (70 mg, 0.1 mmol) and CuI (19 mg, 0.1 mmol) were added. The solution was stirred at room temperature for 16 hours before the solvent was removed *in vacuo*. The residue was purified by silica chromatography eluted by a solvent gradient from neat hexane to neat DCM. **Yield**: 1.00 g (95%). **^{1}H NMR** (600 MHz; CD_2Cl_2): δ_H 7.50 (d, $^3J_{HH}$ = 8.5 Hz, 2H, H_e), 7.46 (d, $^3J_{HH}$ = 8.5 z, 2H, H_g), 7.36 (dd, $^3J_{HH}$ = 3.1 Hz, $^4J_{HH}$ = 2.0 Hz, 1H, H_a), 7.34 (d, $^3J_{HH}$ = 8.4 Hz, 2H, H_d), 7.23 (d, $^3J_{HH}$ = 8.5 Hz, 2H, H_f), 6.25-6.23 (m, 2H, H_b+H_c), 2.51 (s, 3H, H_h), 1.40 (s, 9H, H_i) ppm. **^{13}C{^{1}H} NMR** (150 MHz; CD_2Cl_2): δ_C 149.1, 139.6, 134.3, 134.2, 131.7, 130.5, 129.0, 125.7, 123.0, 121.7, 119.3, 114.7, 110.5, 89.4, 89.3, 83.8, 27.3, 15.0 ppm. **Acc-MS**(ASAP$^+$): *m/z* 390.1552 $[M+H]^+$ calcd. for $C_{24}H_{24}NO_2S$ *m/z* 390.1528 ($|\Delta m/z|$ = 6.2 ppm). **Anal. Calc.** for $C_{24}H_{23}NO_2S{\cdot}\frac{3}{4}H_2O$: C, 71.53; H, 6.13; N, 3.34 %. **Found**: C, 71.51; H, 5.75; N, 3.34 %.

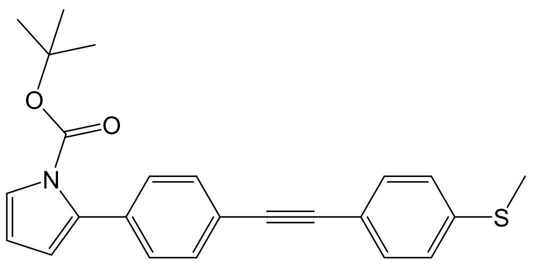

1-hexyl-2-(4-((4-(methylthio)phenyl)ethynyl)phenyl)-1H-pyrrole. NaOMe (0.276 g, 5.12 mmol) was added to a solution containing *tert*-butyl 2-(4-((4-(methylthio)phenyl)ethynyl)phenyl)-1H-pyrrole-1-carboxylate (1.00 g, 2.56 mmol) in THF (50 mL), the solution was heated to reflux for 6 hours before the solvent was cooled and removed in vacuo. The residue was extracted with DCM and water. The organic layer was collected, dried over $MgSO_4$ and filtered. The filtrate was collected and solvent removed *in vacuo* with care not to heat the sample over 40°C. The residue was dissolved in DMF. 1-bromohexane (1.43 mL, 1.68 g, 10.24 mmol) and K_2CO_3 (1.41 g, 10.24 mmol) were added and the solution was stirred for 16 hours at room temperature. The solution was extracted with Et_2O and water, collecting the organic fraction and drying it over $MgSO_4$ before filtration. The filtrate was collected and solvent removed *in vacuo* with care not to heat the sample over 40°C. The residue was purified by silica chromatography eluted by a solvent gradient from neat hexane to DCM:hexane (1:1) to yield a white solid that turned yellow over time. **Yield**: 0.31 g (33 %). **^{1}H NMR** (600 MHz; CD_2Cl_2): δ_H 7.59 (d, $^3J_{HH}$ = 8.2 Hz, 2H, H_e), 7.50 (d, $^3J_{HH}$ = 8.4 Hz, 2H, H_g), 7.41 (d, $^3J_{HH}$ = 8.5 Hz, 2H, H_d), 7.27 (d, $^3J_{HH}$ = 8.4 Hz, 2H, H_f), 6.83 (dd, $^3J_{HH}$ = 2.7 Hz, $^4J_{HH}$ =1.8 Hz, 1H, H_a), 6.24-6.20 (m, 2H, H_b+H_c), 4.01-3.97 (m, 2H, H_i), 2.54 (s, 3H, H_h), 1.70-1.66 (m, 2H, H_j), 1.26-1.21 (m, 6H, H_k+H_l+H_m), 0.87 (t, $^3J_{HH}$ = 7.0 Hz, 3H, H_n) ppm. **^{13}C{^{1}H} NMR** (150 MHz; CD_2Cl_2): δ_C 139.6, 133.7, 133.4, 131.7, 131.4, 128.4, 125.7, 122.8, 121.2, 119.3, 109.2, 107.9, 89.5, 89.3, 47.3, 31.4, 31.2, 26.2, 22.4, 15.0, 13.7 ppm. **Acc-MS**(ASAP$^+$): *m/z* 374.1952 $[M+H]^+$ calcd. for $C_{25}H_{28}NS$ *m/z* 374.1942 ($|\Delta m/z|$ = 2.7 ppm).[iii]

iii Sample too unstable for elemental analysis

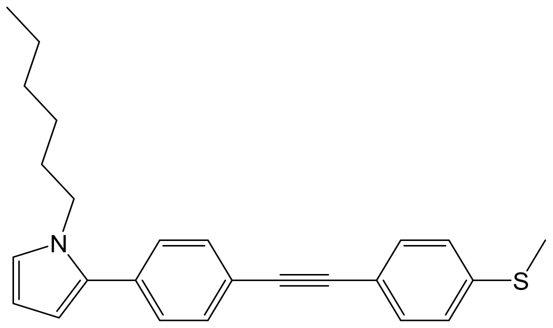

2-(1-hexyl-5-(4-((4-(methylthio)phenyl)ethynyl)phenyl)-1H-pyrrol-2-yl)-4-(1-hexyl-5-(4-((4-(methylthio)phenyl)ethynyl)phenyl)-2H-pyrrol-1-ium-2-ylidene)-3-oxocyclobut-1-en-1-olate

3b. A mixture of 1-hexyl-2-(4-((4-(methylthio)phenyl)ethynyl)phenyl)-1H-pyrrole (0.5 g, 1.34 mmol), and squaric acid (0.076 g, 0.67 mmol) in n-BuOH/Toluene (10 ml:10 ml) was refluxed for 6 h. After removal of the solvent and extraction into DCM the organic phase was washed with water, dried over $MgSO_4$ and filtered. The solvent was removed from the filtrate under reduced pressure to give a blue solid. The product was purified by column chromatography (silica gel, hexane/methylene chloride 1:9) to give a blue solid. **Yield**: 0.15 g (28 %). **^{1}H NMR** (600 MHz; CD_2Cl_2): δ_H 7.92 (d, $^3J_{HH}$ = 4.3 Hz, 2H, H_a), 7.66 (d, $^3J_{HH}$ = 8.7 Hz, 4H, H_c), 7.53 (d, $^3J_{HH}$ = 8.4 Hz, 4H, H_e), 7.48 (d, $^3J_{HH}$ = 8.7 Hz, 4H, H_d), 7.25 (d, $^3J_{HH}$ = 8.4 Hz, 4H, H_f), 6.64 (d, $^3J_{HH}$ = 4.3 Hz, 2H, H_b), 4.85 (t, $^3J_{HH}$ = 7.4 Hz, 2H, H_h), 2.52 (s, 6H, H_g), 1.59-1.55 (m, 4H, H_i),[iv] 1.19-1.10 (m, 12H, H_j+H_k+H_l), 0.79 (t, $^3J_{HH}$ = 7.2 Hz, 6H, H_m) ppm.[v] **Acc-MS**(ASAP$^+$): *m/z* 799.3393 $[M+H]^+$ calcd. for $C_{54}H_{55}N_2O_2S_2$ *m/z* 827.3705 ($|\Delta m/z|$ = 1.3 ppm). **Anal. Calc.** for $C_{54}H_{52}N_2O_2S_2 \cdot ⅓CH_2Cl_2$: C, 76.49; H, 6.22; N, 3.28 %. **Found**: C, 76.25; H, 6.21; N, 3.36 %.

---

iv Solvent peak overlap

v Solubility too low for ^{13}C NMR

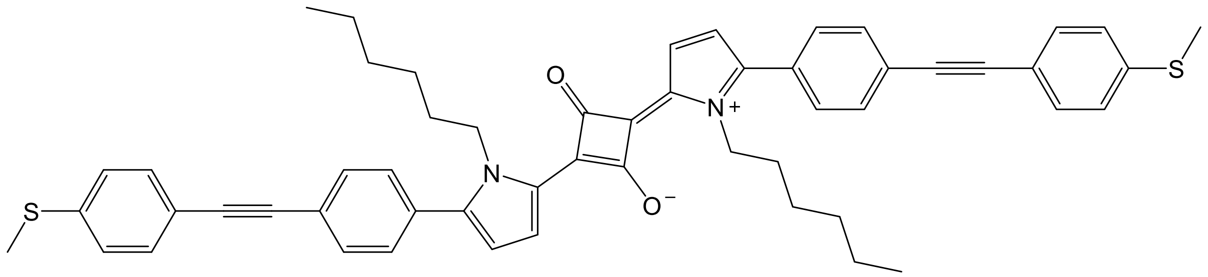

O
N
N+
O−
S
S

S2. NMR spectra of reported compounds

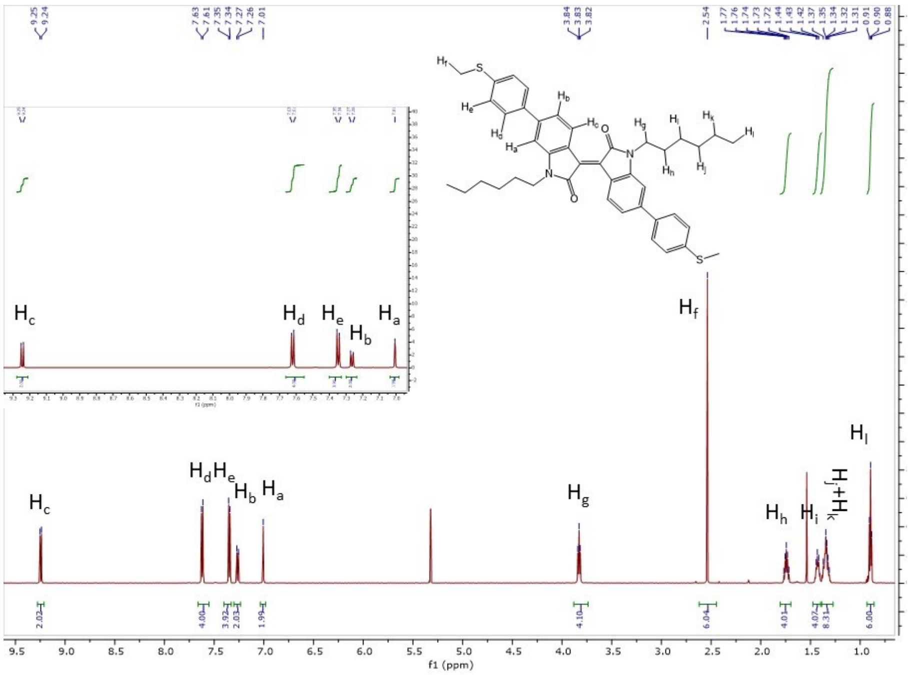

Figure S1. ^{1}H NMR spectrum of **1a** recorded in CD_2Cl_2.

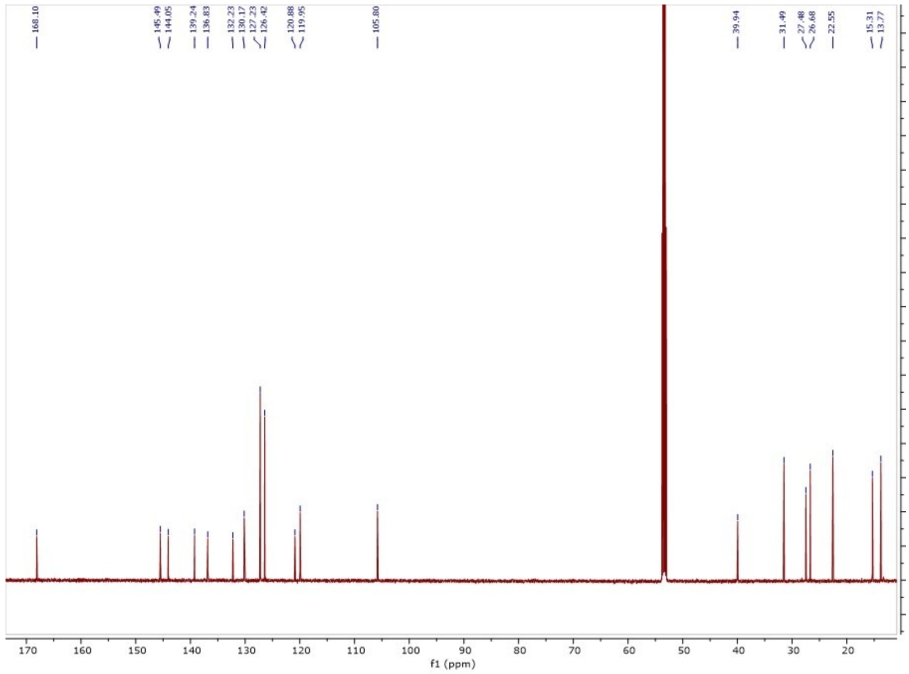

Figure S2. ^{13}C NMR spectrum of **1a** recorded in CD_2Cl_2.

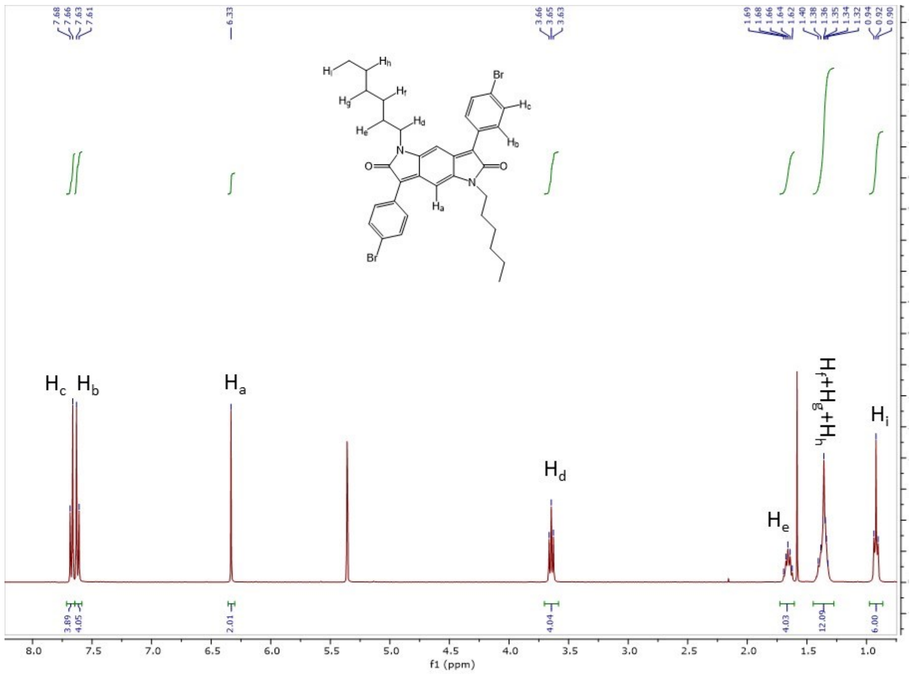

Figure S3. ^{1}H NMR spectrum of 3,7-bis(4-bromophenyl)-1,5-dihexylpyrrolo[2,3-f]indole-2,6-dione recorded in CD_2Cl_2.

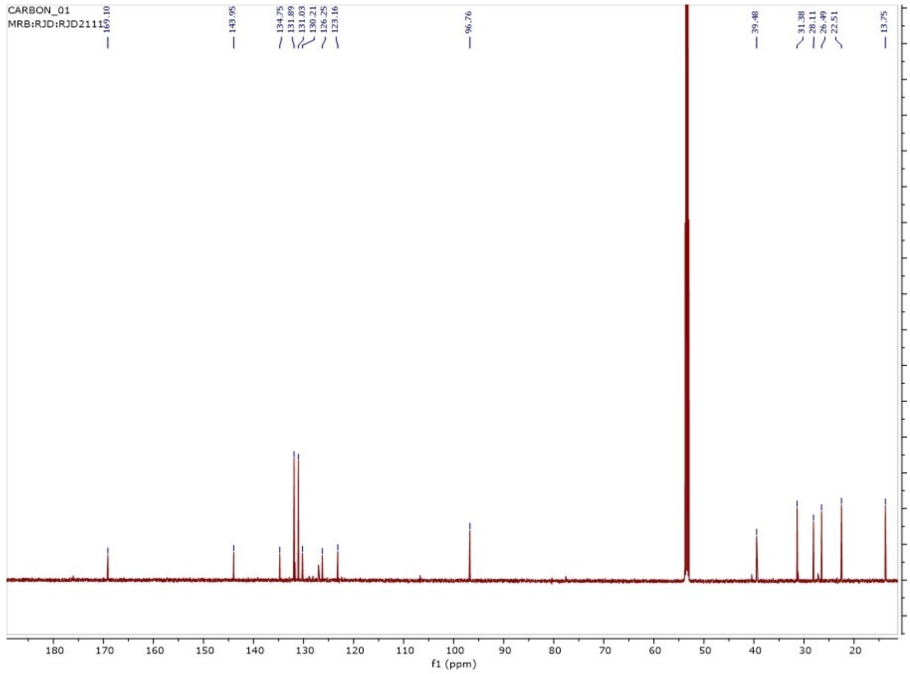

Figure S4. ^{13}C NMR spectrum of 3,7-bis(4-bromophenyl)-1,5-dihexylpyrrolo[2,3-f]indole-2,6-dione recorded in CD_2Cl_2.

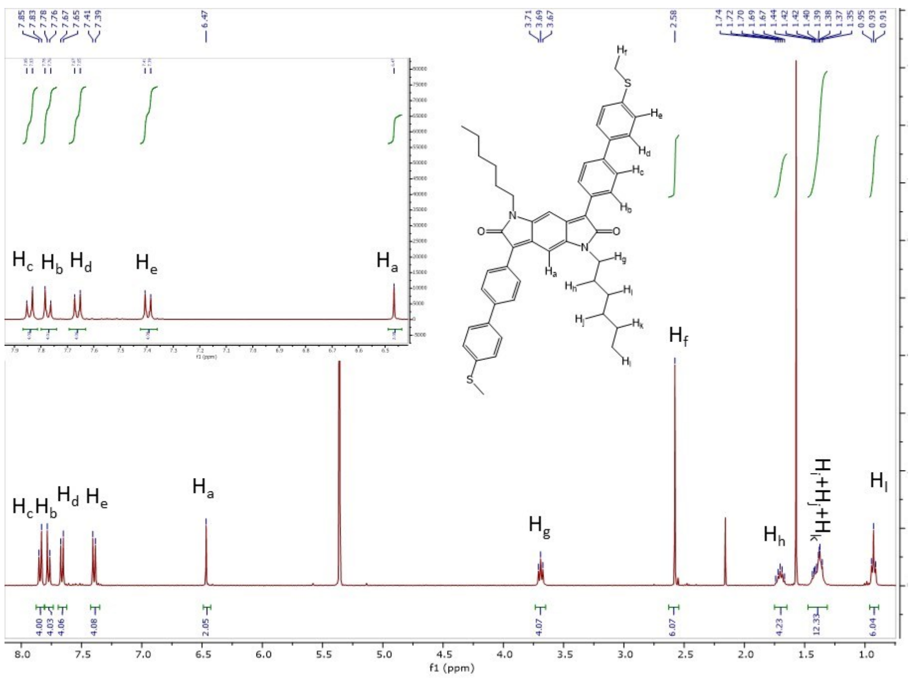

Figure S5. ^{1}H NMR spectrum of **2a** recorded in CD_2Cl_2.

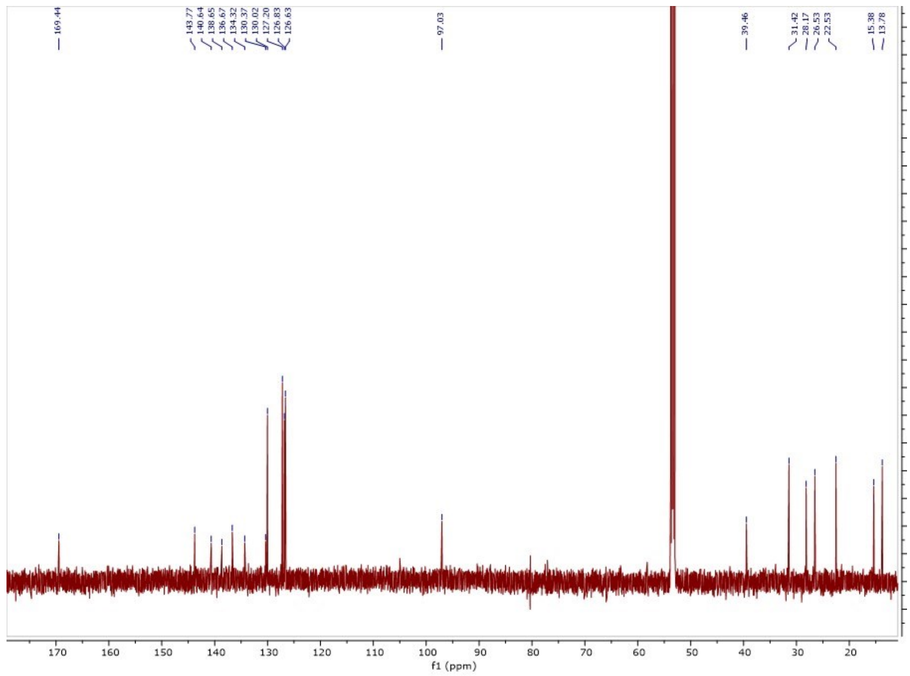

Figure S6. ^{13}C NMR spectrum of **2a** recorded in CD_2Cl_2.

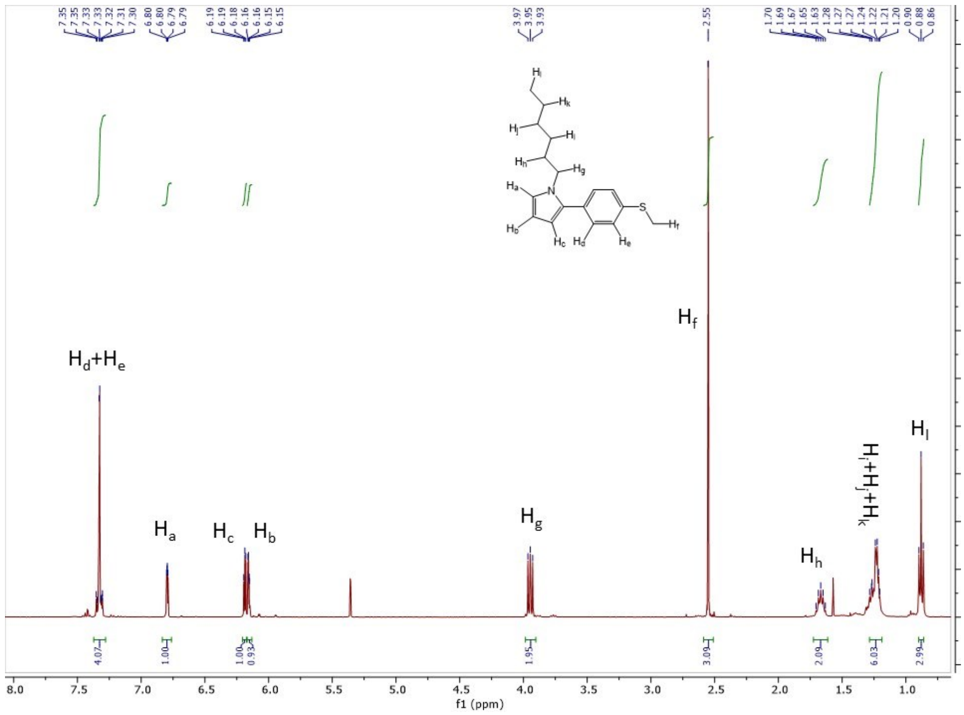

Figure S7. ^{1}H NMR spectrum of 1-hexyl-2-(4-(methylthio)phenyl)-1H-pyrrole recorded in CD_2Cl_2.

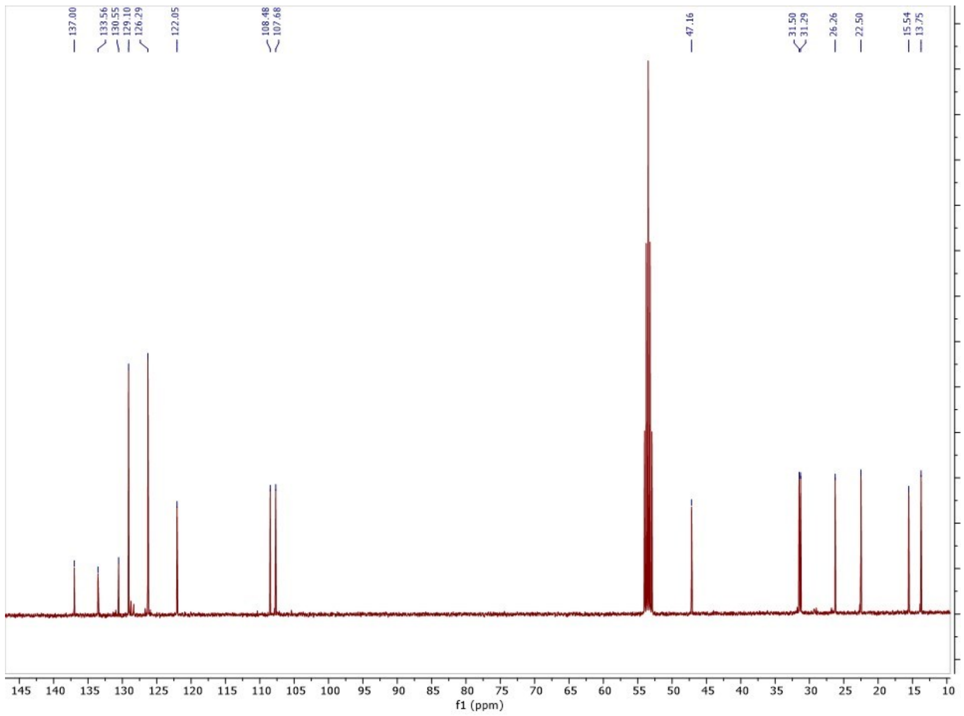

Figure S8. ^{13}C NMR spectrum of 1-hexyl-2-(4-(methylthio)phenyl)-1H-pyrrole recorded in CD_2Cl_2.

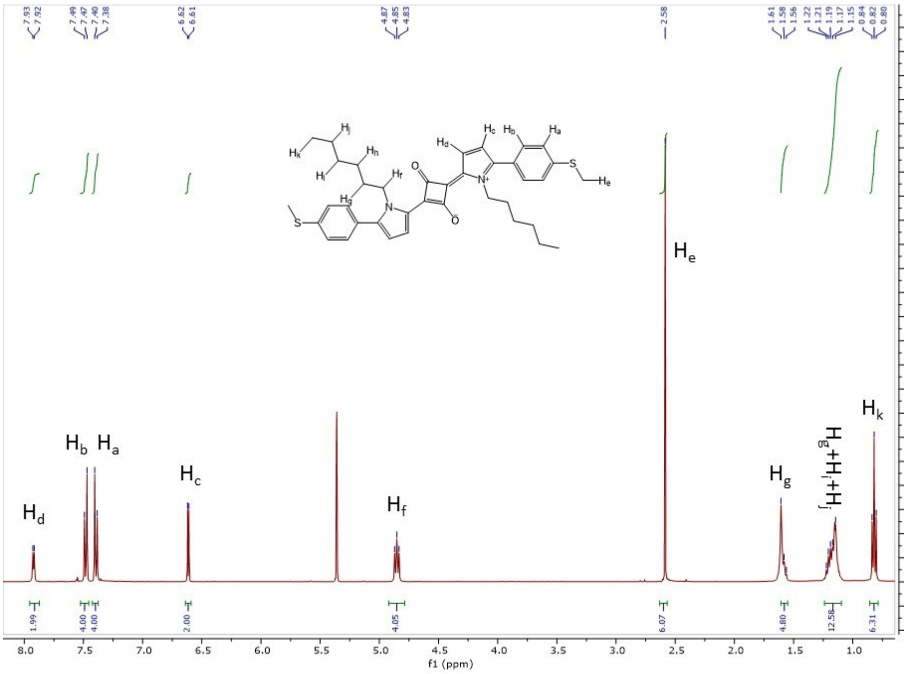

Figure S9. ^{1}H NMR spectrum of **3a** recorded in CD_2Cl_2.

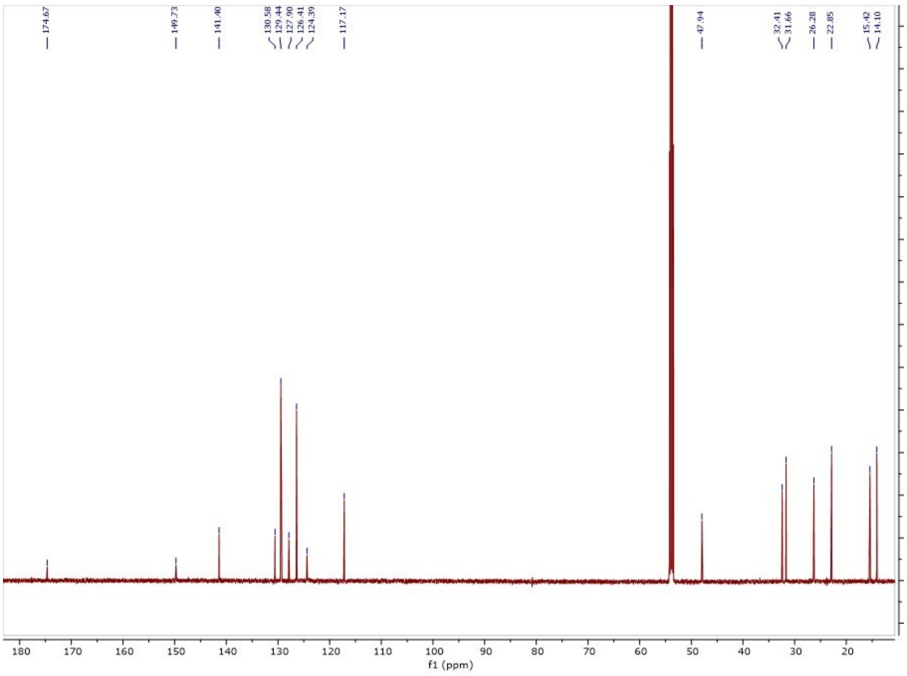

Figure S10. ^{13}C NMR spectrum of **3a** recorded in CD_2Cl_2.

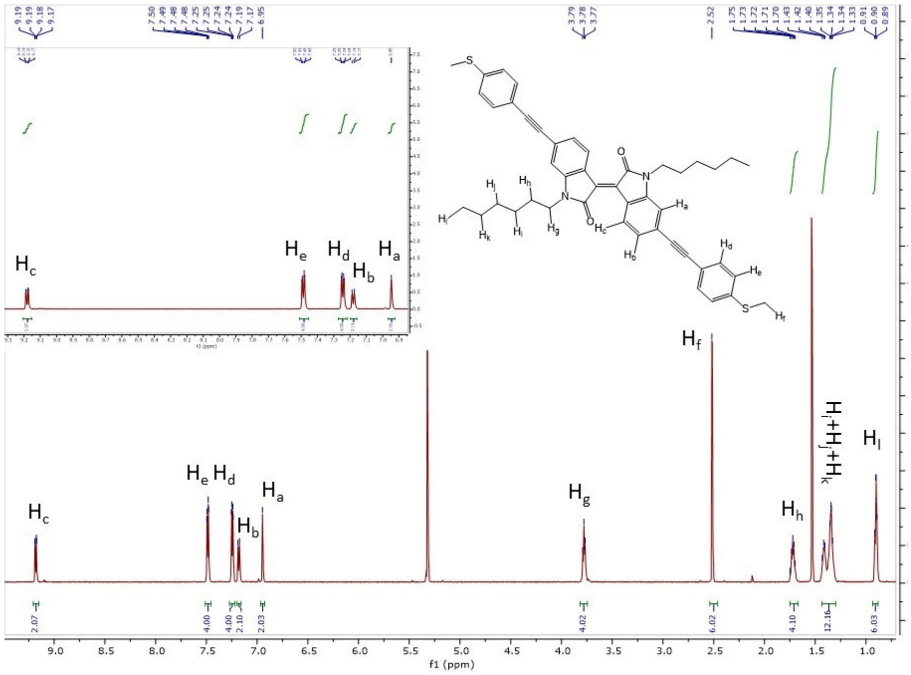

Figure S11. ^{1}H NMR spectrum of **1b** recorded in CD_2Cl_2.

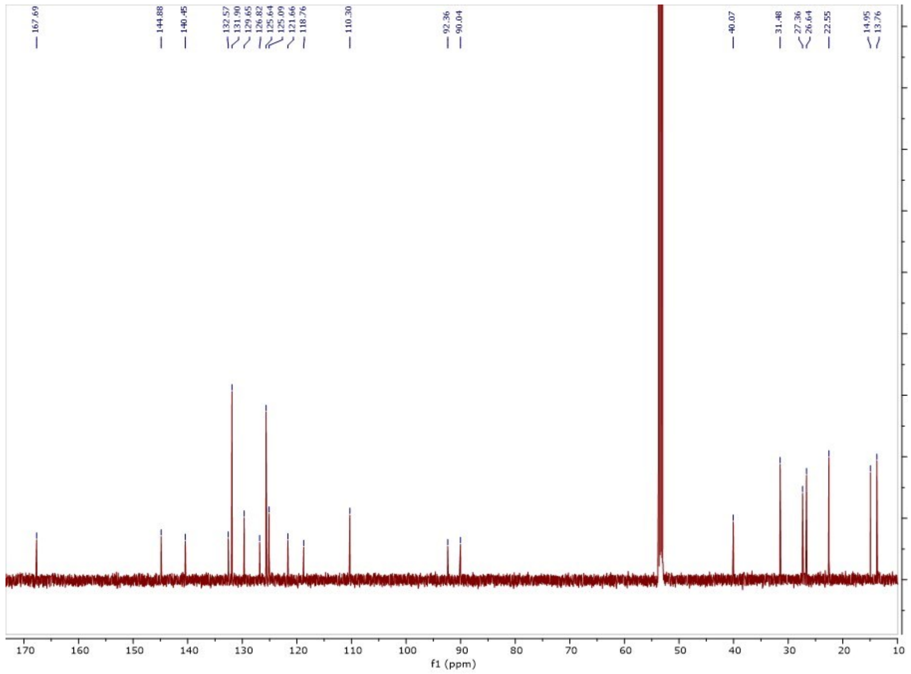

Figure S12. ^{13}C NMR spectrum of **1b** recorded in CD_2Cl_2.

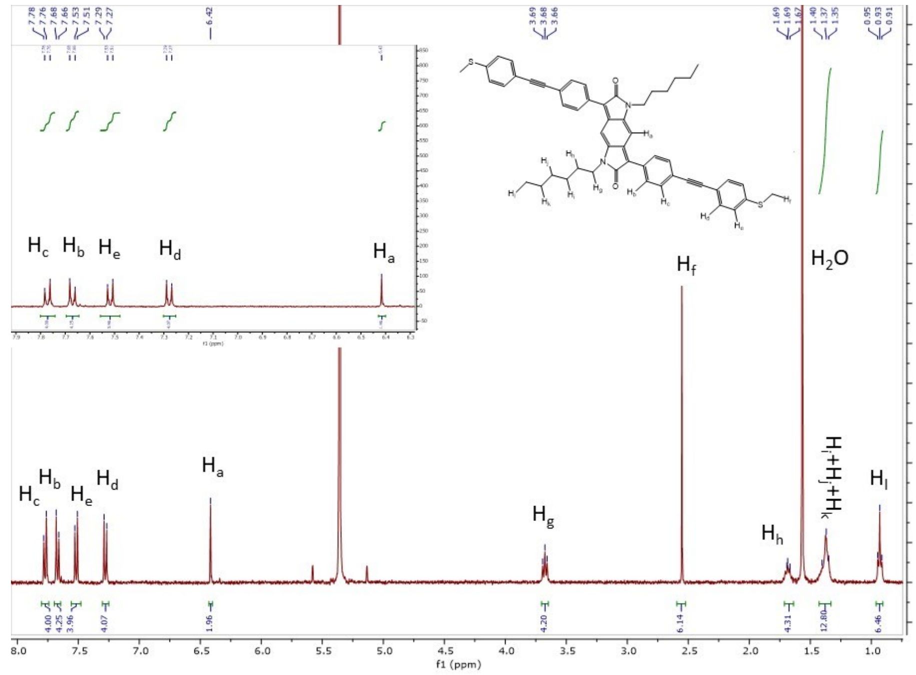

Figure S13. ^{1}H NMR spectrum of **2b** recorded in CD_2Cl_2.

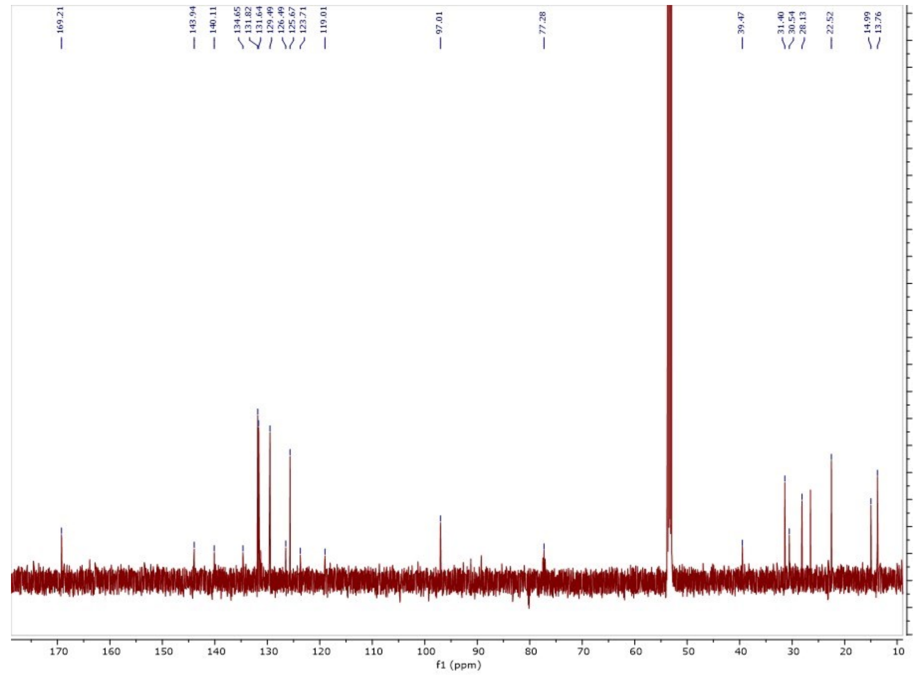

Figure S14. ^{13}C NMR spectrum of **2b** recorded in CD_2Cl_2.

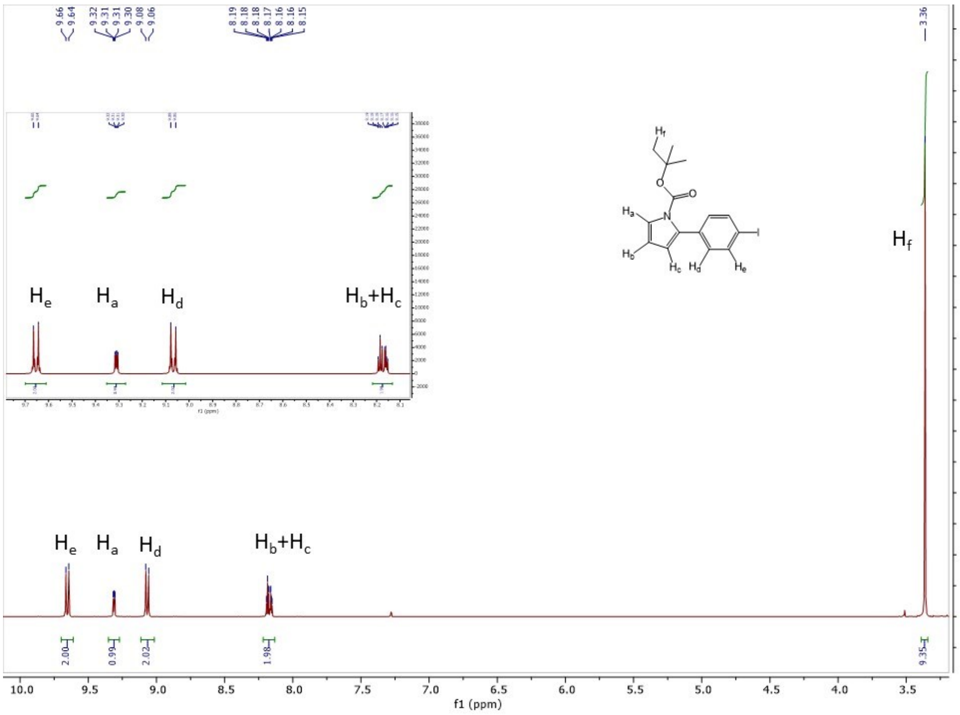

Figure S15. ^{1}H NMR spectrum of tert-butyl 2-(4-iodophenyl)-1H-pyrrole-1-carboxylate recorded in CD_2Cl_2.

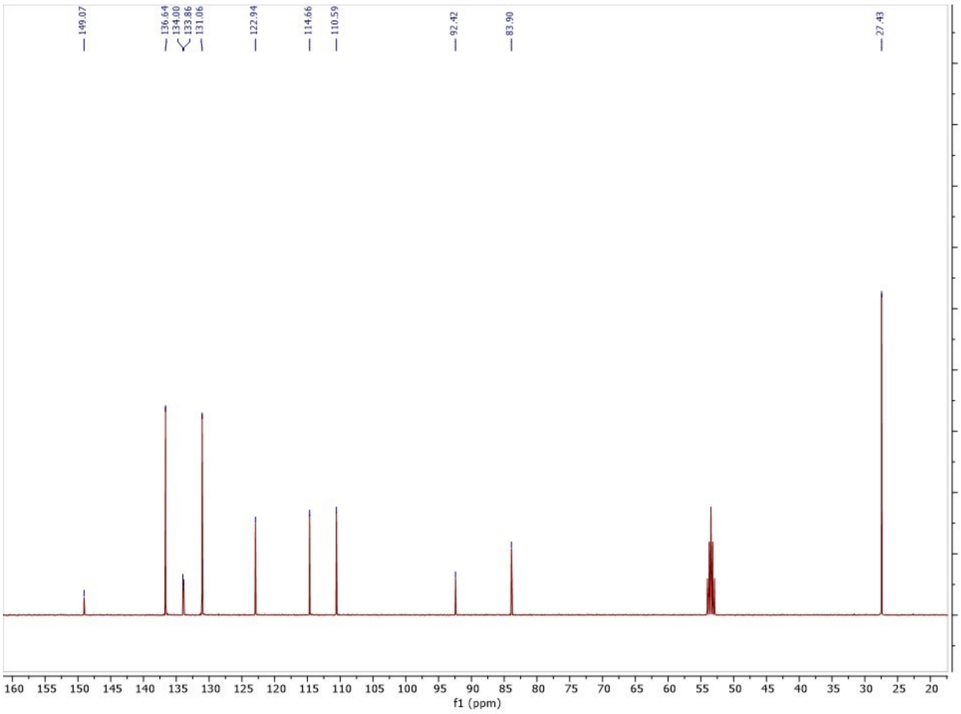

Figure S16. ^{13}C NMR spectrum of tert-butyl 2-(4-iodophenyl)-1H-pyrrole-1-carboxylate recorded in CD_2Cl_2.

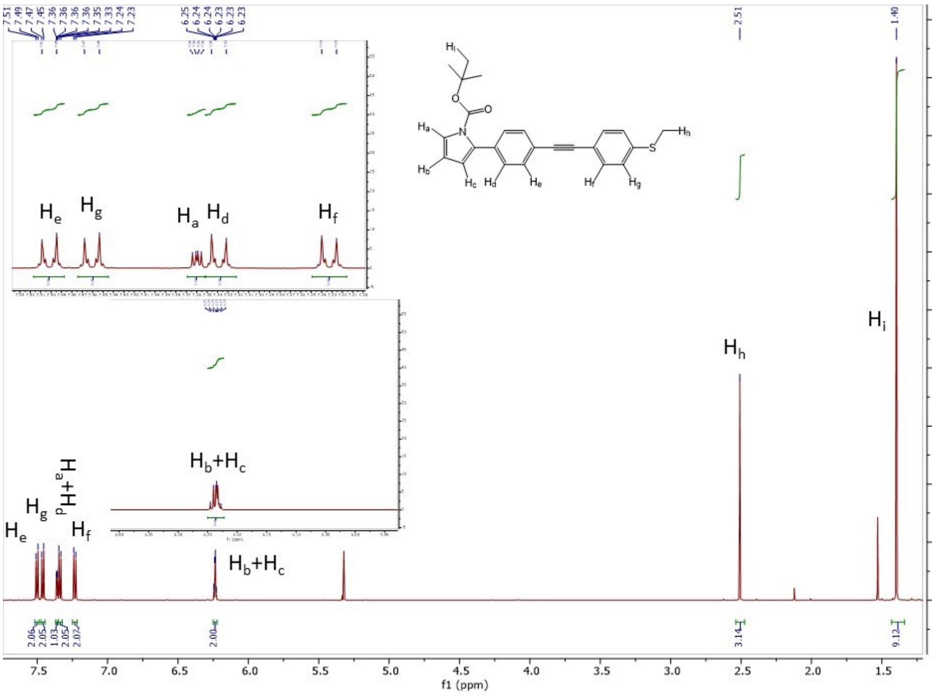

Figure S17. ^{1}H NMR spectrum of tert-butyl 2-(4-((4-(methylthio)phenyl)ethynyl)phenyl)-1H-pyrrole-1-carboxylate recorded in CD_2Cl_2.

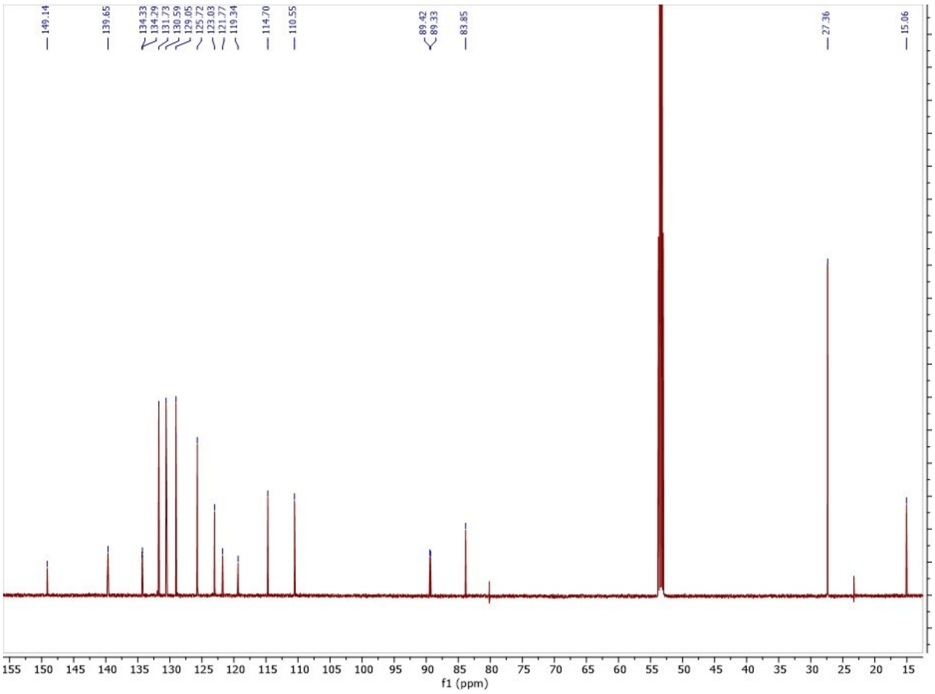

Figure S18. ^{13}C NMR spectrum of tert-butyl 2-(4-((4-(methylthio)phenyl)ethynyl)phenyl)-1H-pyrrole-1-carboxylate recorded in CD_2Cl_2.

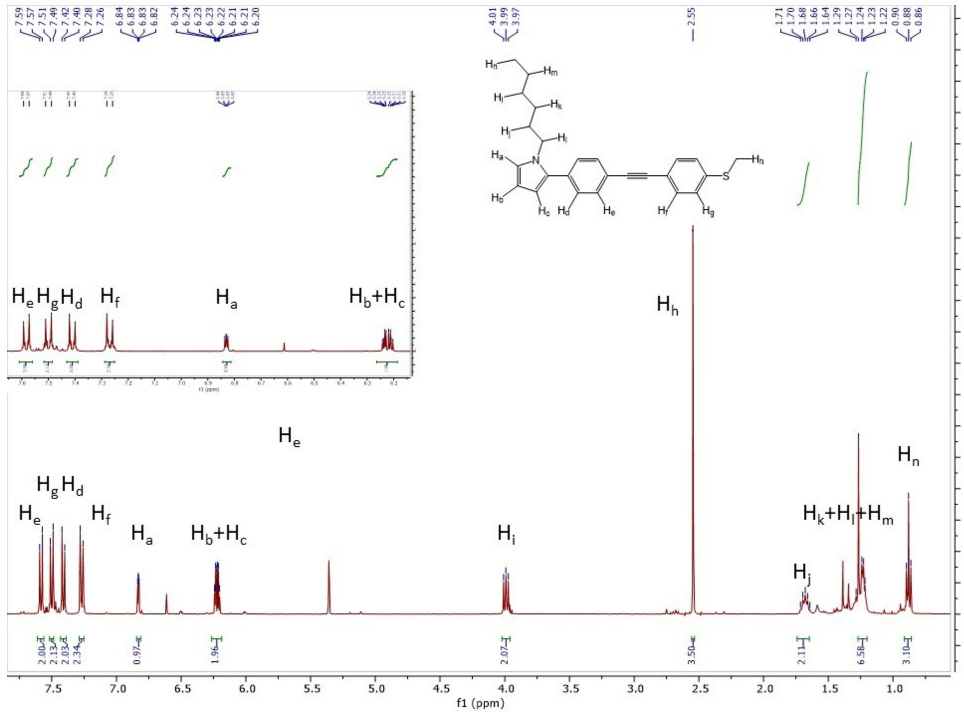

Figure S19. ^{1}H NMR spectrum of 1-hexyl-2-(4-((4-(methylthio)phenyl)ethynyl)phenyl)-1H-pyrrole recorded in CD_2Cl_2.

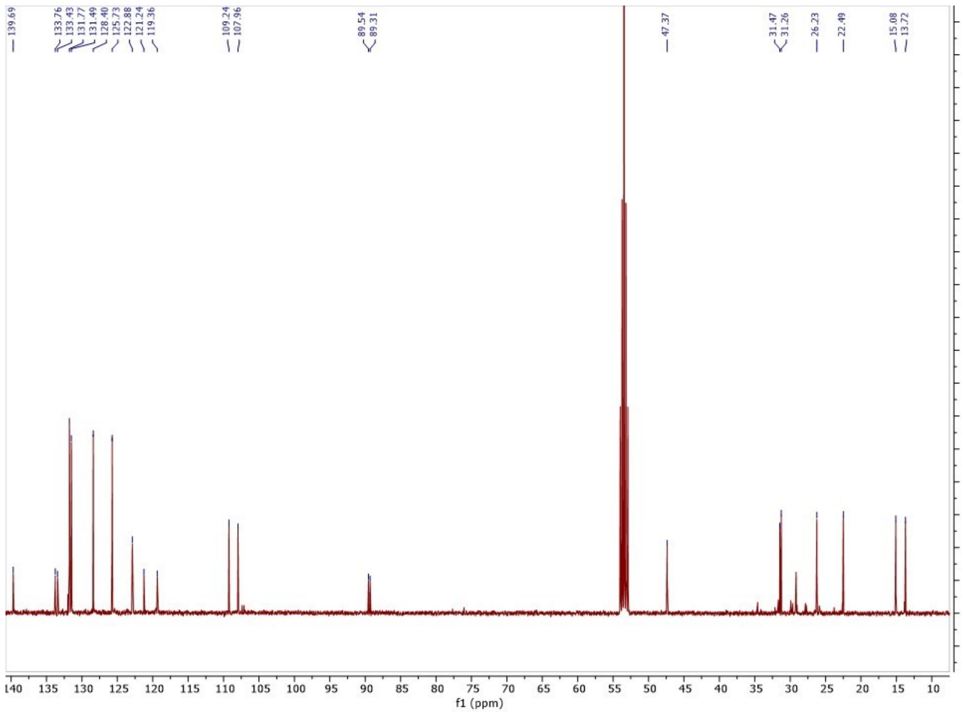

Figure S20. ^{13}C NMR spectrum of 1-hexyl-2-(4-((4-(methylthio)phenyl)ethynyl)phenyl)-1H-pyrrole recorded in CD_2Cl_2.

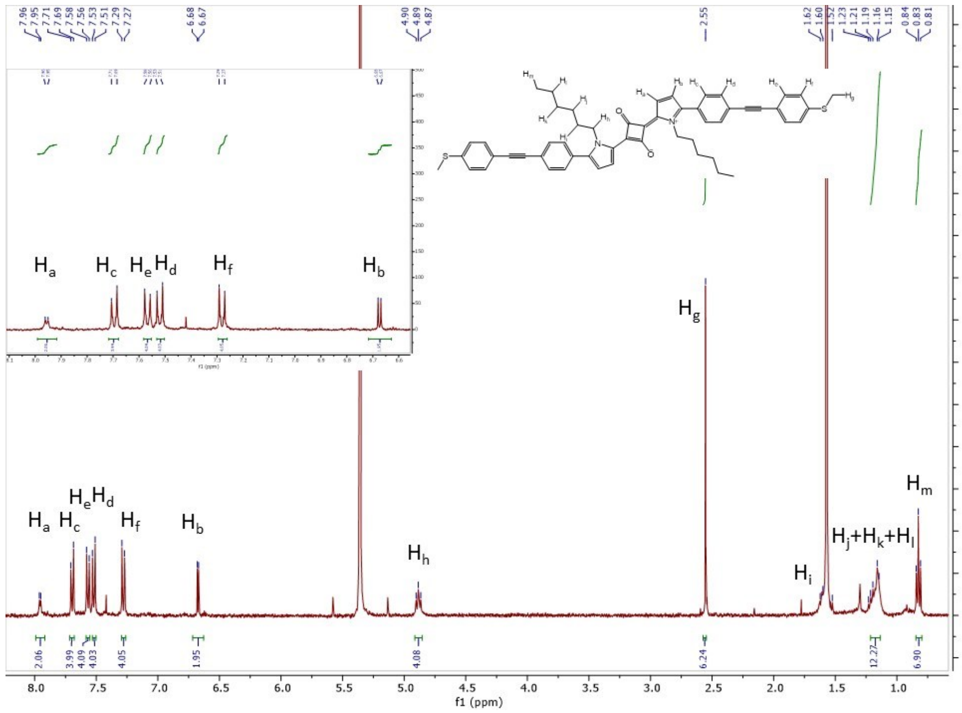

Figure S21. ^{1}H NMR spectrum of **3b** recorded in CD_2Cl_2.

S3. X-ray Crystallography

Single-crystal X-ray diffraction experiments for **1a** and **2b** were carried out on a Bruker D8 Venture 3-circle diffractometer, equipped with a CPA area detector PHOTON III C14 MM, using Mo-*K*α (**1b**) or Cu-*K*α (**2b**) radiation from Incoatec IμS 3.0 microsources with focusing mirrors. The crystals were cooled using Cryostream 700 open-flow N_2 gas cryostat (Oxford Cryosystems). The data were collected in shutterless mode by narrow frame ω scans covering full sphere of reciprocal space, using APEX3 v.2017.3-0 software, reflection intensities integrated using SAINT v8.40A software (Bruker AXS, 2019). Data were corrected for absorption by semi-empirical method based on Laue equivalents and multiple scans using SADABS 2016/2 software.[5] Both structures were solved by dual-space intrinsic phasing, using SHELXT 2018/2 program[6] and refined by full-matrix least squares using SHELXL 2018/3 software[6] on Olex2 platform.[7] Crystal data and other experimental details are listed in Table S1, molecular structures are shown in Fig. S22. In both structures the molecule has crystallographic inversion symmetry. In **1b** the bis(ethynyl)diindolidene-dione moiety is approximately planar (with the mean deviation of 0.1 Å), resulting in a short intramolecular contact C(15^1)-H...O=C(8) (C-H 0.96(2), H...O 2.08(2) Å), while the phenyl planes are inclined to this moiety by 22°. In **2b** the central fused system is planar (mean deviation 0.02 Å), the mean line of the diareneethyne rod is inclined to the latter plane by 9°. The two arene rings in the rod are coplanar within experimental error and inclined to the central moiety by 28°. The S…S distances for **1b** and **2b** are 26.8076(10) and 30.023(2) Å respectively.

Table S1. Crystal data and structure refinement for structures **1b** and **2b**.

	**1b**	**2b**
CCDC dep.number	2301298	2301299
Empirical formula	$C_{46}H_{46}N_2O_2S_2$	$C_{52}H_{50}N_2O_2S_2$
Formula weight	722.97	799.06
Temperature/K	120	200*
Crystal system	monoclinic	triclinic
Space group	$P2_1/c$ (no. 14)	$P\bar{1}$ (no. 2)
a/Å	15.8320(4)	5.8649(5)
b/Å	6.7598(2)	8.7643(7)
c/Å	17.9453(4)	22.1127(19)
α/°	90	91.935(7)
β/°	99.6745(10)	95.986(6)
γ/°	90	107.642(6)
Volume/Å^3	1893.21(9)	1074.70(16)
Z	2	1
ρ_{calc}, g/cm^3	1.268	1.235
μ/mm^{-1}	0.182	1.452
F(000)	768	424
Radiation	Mo Kα (λ = 0.71073)	Cu Kα (λ = 1.54184)
Reflections collected	29652	9489
Independent reflections, R_{int}	6020 [R_{int} = 0.0514, R_σ = 0.0438]	3624 [R_{int} = 0.0723, R_σ = 0.0728]
Data/restraints/parameters	6020/0/327	3624/3/277
Goodness-of-fit on F^2	1.027	1.052
Final R_1 indexes [$I \geq 2\sigma(I)$]	R_1 = 0.0474, wR_2 = 0.1079	R_1 = 0.0780, wR_2 = 0.2098
Final wR_2 indexes [all data]	R_1 = 0.0647, wR_2 = 0.1166	R_1 = 0.1105, wR_2 = 0.2369

Largest diff. peak/hole / e Å^{-3}	0.55/-0.33	0.47/-0.56

* Between 180 and 170 K a reversible transition into an incommensurately modulated phase occurs

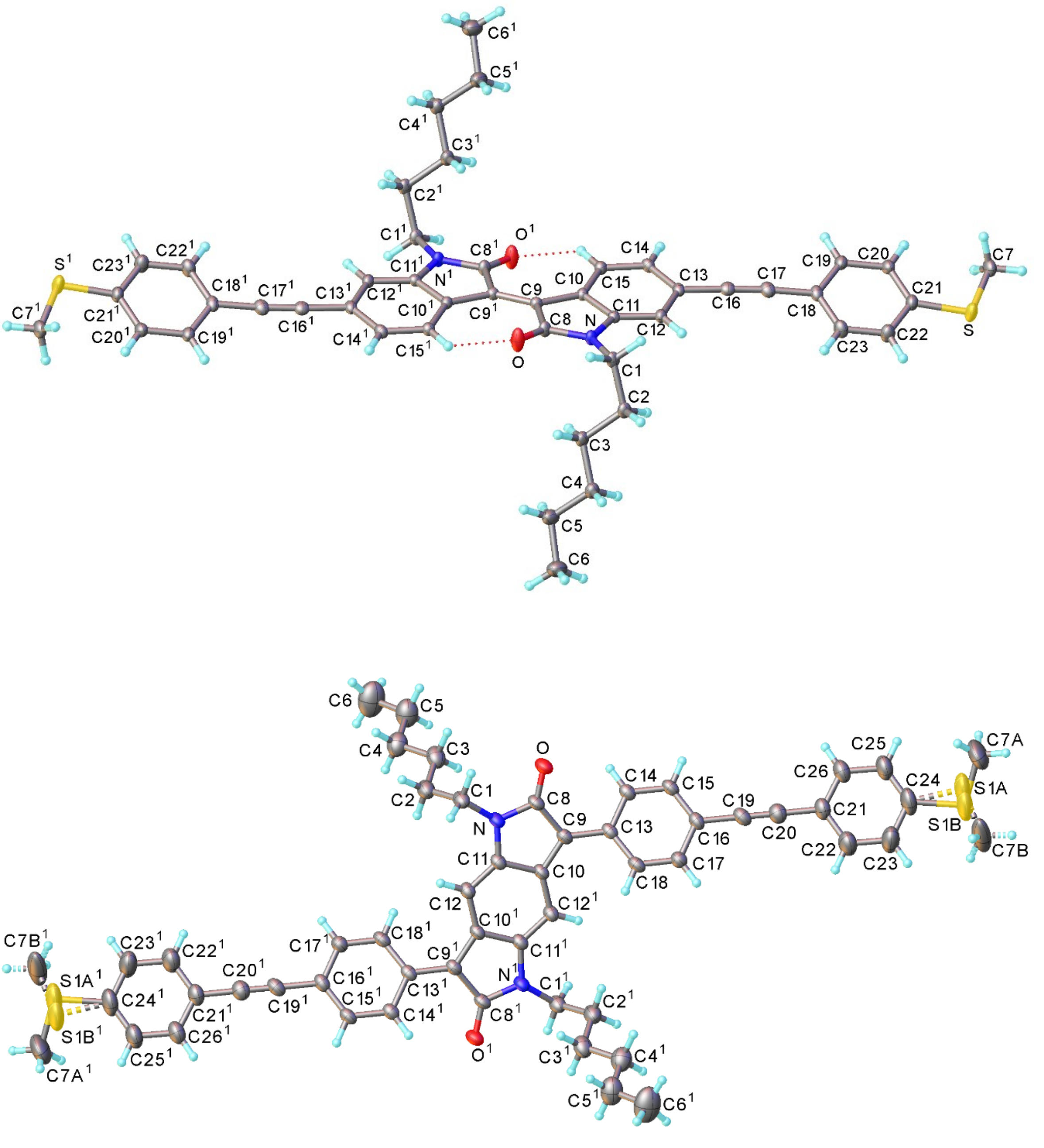

Figure S22. X-ray molecular structures of **1b** (top) and **2b** (bottom, showing the disorder of SMe group in a 0.5:0.5 ratio). Thermal ellipsoids are drawn at the 50% probability level. Primed atoms are generated by the inversion centers.

S4. Photophysical measurements

UV-visible absorption spectra were recorded at room temperature in DCM using an Evolution 220 UV-visible spectrophotometer (Thermo Scientific) in quartz cuvettes with a path length of 1 cm. Each of the compounds displayed a HOMO–LUMO gap ranging from 1.85 eV (**3b**) to 2.12 eV (**2a**) (Figure S23 and Table S2). A slight reduction in the HOMO–LUMO gap was observed as the conjugation length of the molecules was extended which is most significantly demonstrated by comparing **1a** (1.92 eV) to **1b** (1.88 eV).

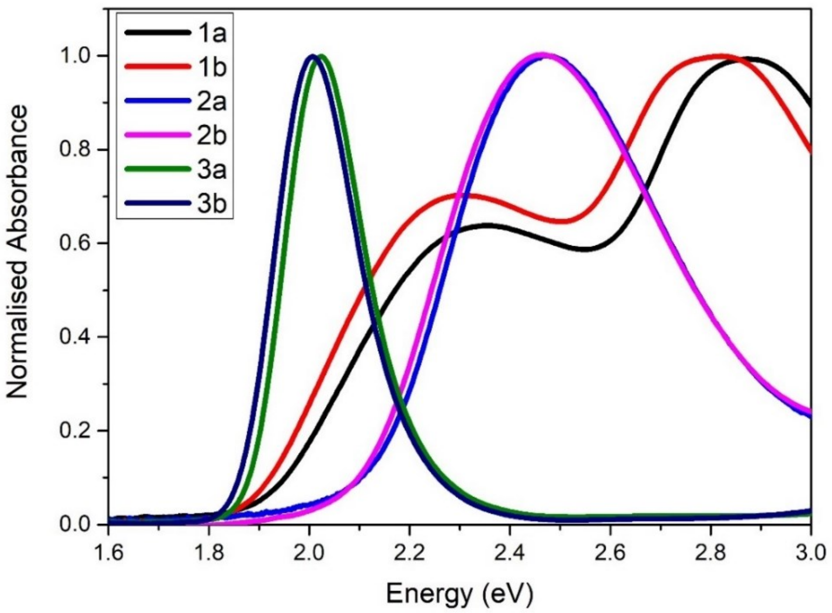

Figure S23. Electronic absorbance of **1a, 2a, 3a, 1b, 2b,** and **3b** recorded in DCM.

Table S2. ΔE|HOMO-LUMO| of compounds **1a, 2a, 3a, 1b, 2b,** and **3b** determined by electronic absorbance.

Compound	ΔE|HOMO-LUMO| (eV)
**1a**	1.92
**1b**	1.88
**2a**	2.12
**2b**	2.10
**3a**	1.87
**3b**	1.85

S5. Stability of bis(pyrrol-2-yl)squaraines 3a and 3b

To examine the potential decomposition of bis(pyrrol-2-yl)squaraine compounds **3a** and **3b**, the effects of solution, light irradiation, surface deposition, and a combination of surface deposition and light irradiation were systematically investigated.

Solution stability

To facilitate the interpretation of the ^{1}H NMR data, only **3a** was subjected to the solution stability study. A solution of **3a** in CD_2Cl_2 was prepared and stored in the dark for one week. ^{1}H NMR spectra were collected regularly over a one-week period to assess if any additional species were formed. Figure S24 displays a comparison of the ^{1}H NMR spectra over this period demonstrating no additional signals, indicating that **3a** is stable in solution and it is a reasonable assumption that **3b** is also stable.

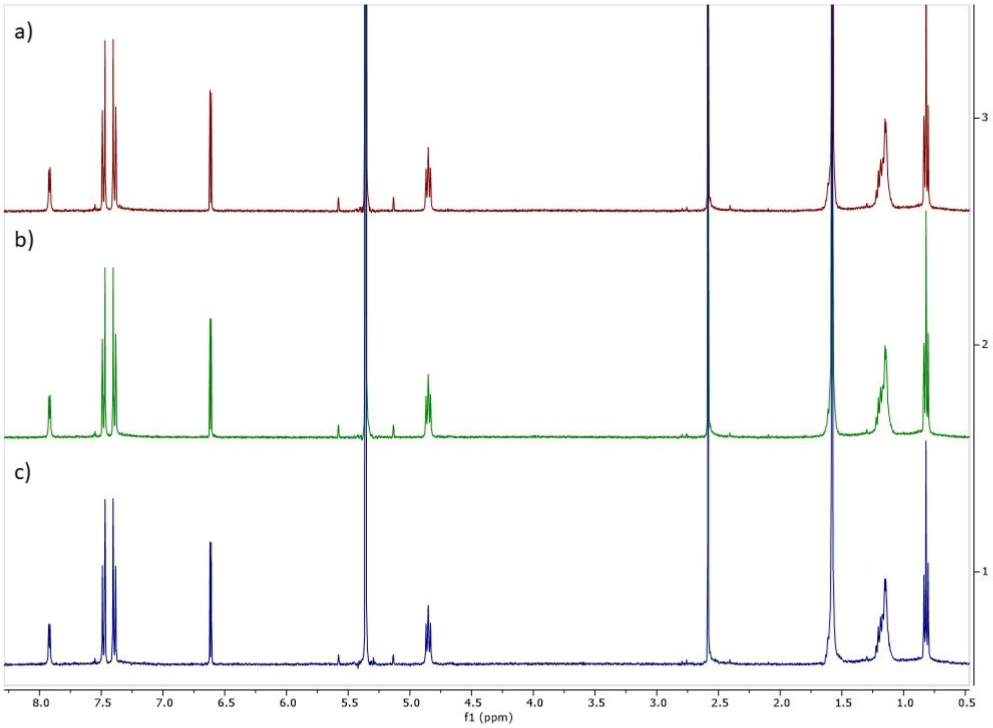

Figure S24. ^{1}H NMR spectra of **3a** in CD_2Cl_2 recorded after a) 1, b) 3, and c) 7 days, stored in the dark.

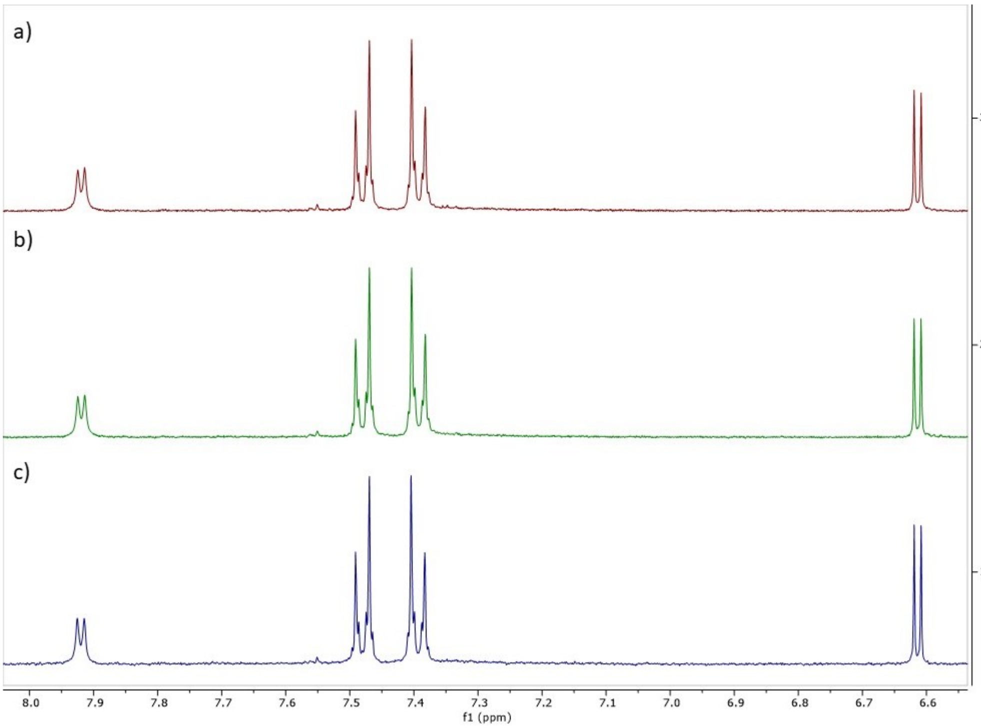

Figure S25. Expansion of the aromatic region of the ^{1}H NMR spectra of **3a** in CD_2Cl_2 recorded over a) 1, b) 3, and c) 7 days, stored in the dark.

Photostability

To examine the photostability of the bis(pyrrol-2-yl)squaraines, a solution of **3a** in CD_2Cl_2 was irradiated by a xenon lamp (300 W) for 15 minutes. As shown in Figure S26, no additional signals were observed in the ^{1}H NMR spectrum after photoirradiation, confirming the photostability of **3a** under any lighting conditions the sample is likely to be exposed to during the course of STM measurements.

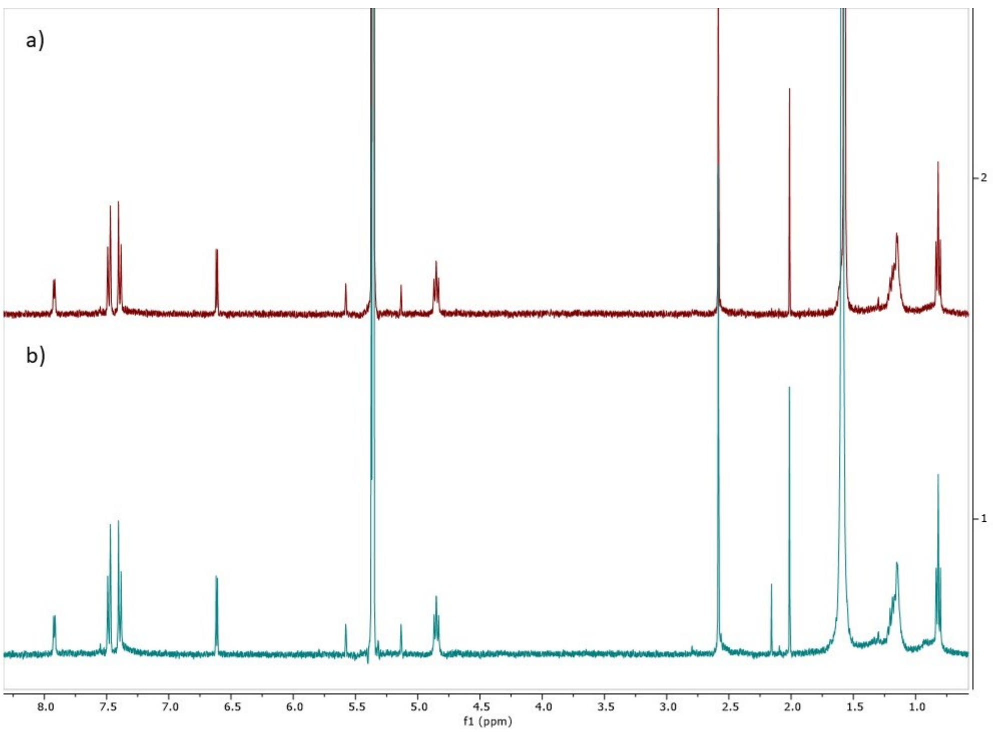

Figure S26. ^{1}H NMR spectra of **3a** in CD_2Cl_2 recorded a) before irradiation and b) after irradiation.

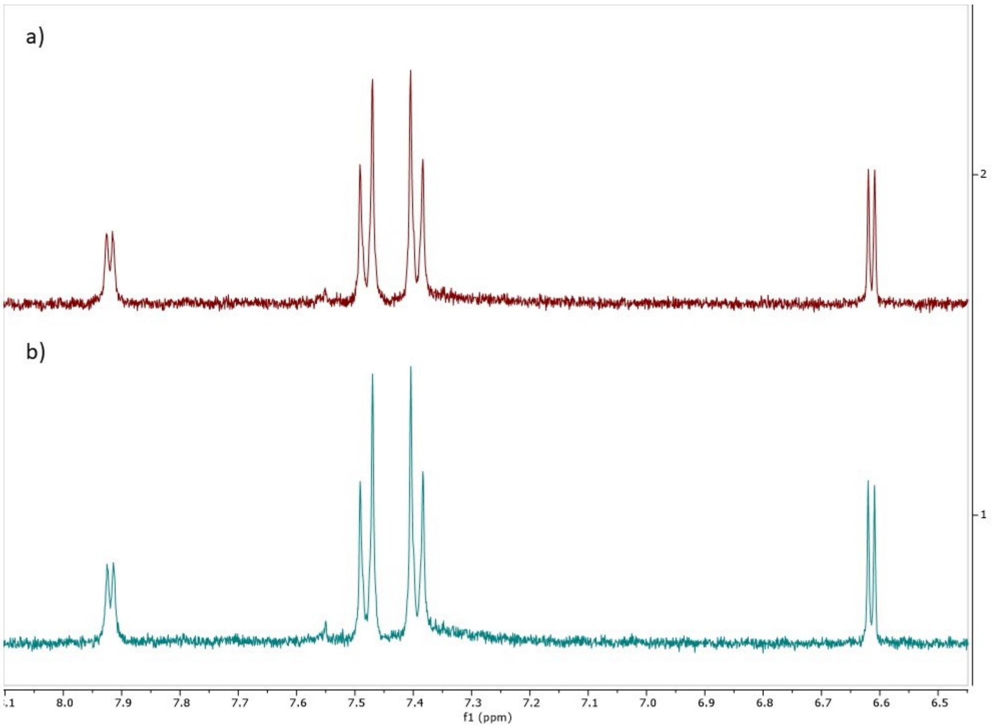

Figure S27. Expansion of the aromatic region of the ^{1}H NMR spectra of **3a** in CD_2Cl_2 recorded a) before irradiation and b) after irradiation.

B) Conductance and Seebeck measurements

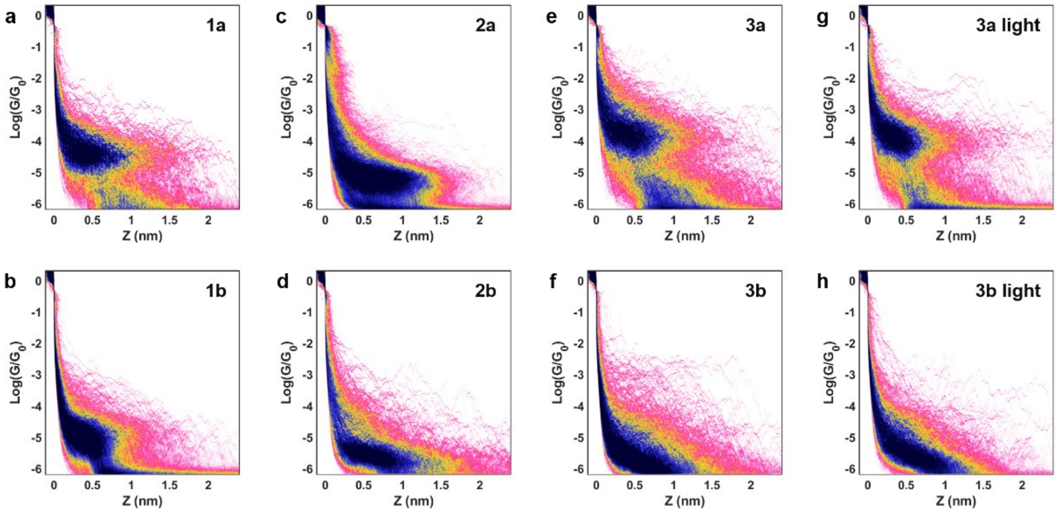

Figure S28. Conductance G vs distance Z histograms of compounds (a) **1a**, (b) **1b**, (c) **2a**, (d) **2b**, (e) **3a** and (f) **3b** in dark, and (g) **3a** and (h) **3b** in light, built with the selected traces that show a molecular response.

To confirm that the longer molecules **1b**, **2b**, and **3b** conducted in a coherent tunnelling regime, their conductance was predicted using the conductance of the shorter molecules **1a**, **2a**, **3a** and accounting for the conductance attenuation due to tunnelling using Equation S1, where G_l is the conductance of the longer molecules **1b**, **2b**, and **3b**, G_s is the conductance of the shorter analogues **1a**, **2a**, **3a**, β is the tunnelling attenuation factor (assumed to be that of oligo(phenylene ethynylene) = 3.4 nm^{-1}) [8] and Δr is the difference in length between the shorter and longer molecules. The values in Table S3 show the conductance values of the longer molecules are reasonably predicted by only accounting for the tunnelling attenuation due to the increased conjugation length of the molecules, Eq. S1.

$$G_l = G_s e^{-\beta \Delta r} \quad \textbf{(Eq. S1)}$$

Table S3. Empirically predicted conductance values of **1b**, **2b**, and **3b**

Molecule	Conductance (log(G/G$_0$)	
	Predicted	Measured
**1b**	-5.1	-5.0
**2b**	-5.9	-5.6
**3b**	-5.6	-5.8

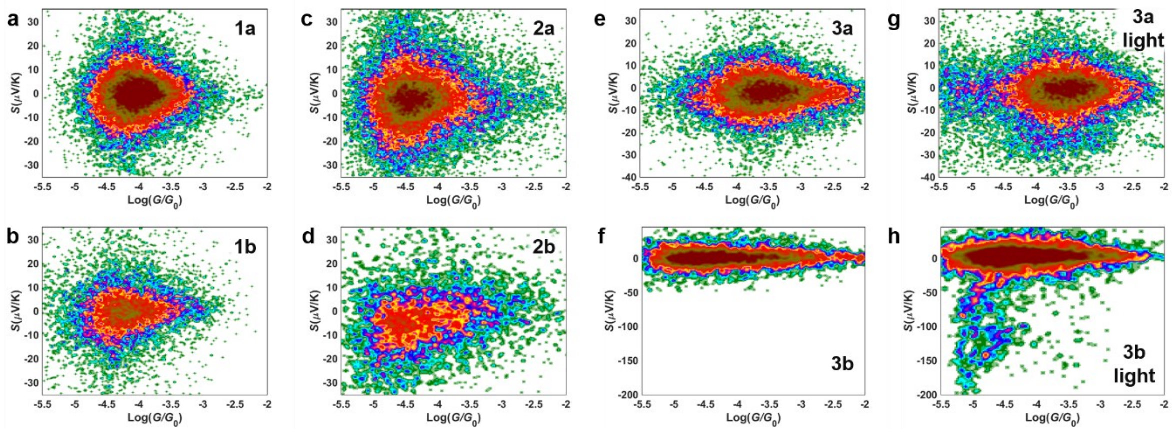

Figure S29. Seebeck coefficient *S* vs Conductance *G* histograms of compounds (a) **1a**, (b) **1b**, (c) **2a**, (d) **2b**, (e) **3a** and (f) **3b** in dark, and (g) **3a** and (h) **3b** in light, built with the temperature difference dependence of the thermovoltage data points measured in the selected traces that show a molecular response.

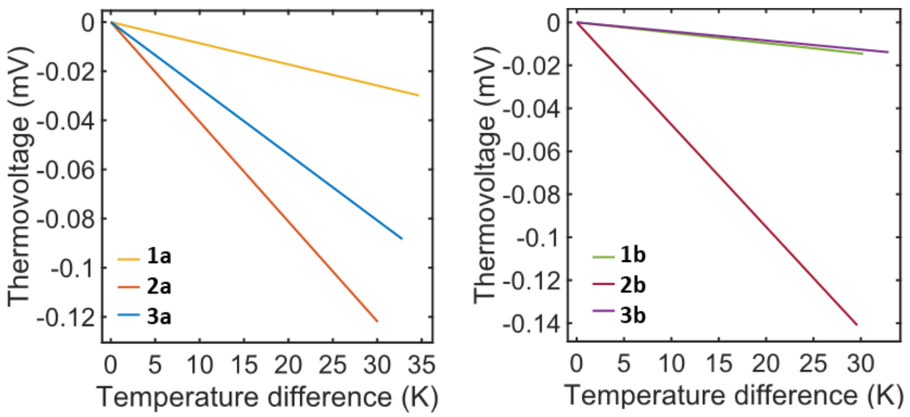

Figure S30. Linear regressions of the temperature difference dependence of the thermovoltage measurements for short "**a**" (Left) and long "**b**" (Right) counterparts of compounds **1**, **2** and **3**. The slope represents the Seebeck coefficient.

The thermoelectric measurements of BPS compounds (**3a** and **3b**) proved to be fickler than the IGG (**1a** and **1b**) and BDP (**2a** and **2b**) compounds. When measuring the Seebeck coefficient of the BPS compounds (**3a** and **3b**) under standard conditions (as previously described), the most probable Seebeck values were -0.7 and +2.6 µV/K for **3a** and **3b**, respectively, where compound **3b** shows HOMO-dominated transport. Besides, additional features were observed as depicted in Figure S31e-f distributed around two different values for both compounds: around -7 and -23 µV/K for compound **3a**, and around -75 and -117 µV/K for compound **3b**.

It was noticed that the most probable measured Seebeck values varied over days ranging from below -3 towards 0 µV/K for compound **3a**, and below 0 to above 3 µV/K for compound **3b**; in both cases a tendency towards more positive values was observed. New samples were prepared and kept in the dark inside the measurement system. When measuring the most probable Seebeck coefficient for some days, these samples showed small variations around -2 and -3 µV/K for compound **3a**, and around 0 and -1 µV/K for compound **3b**. Then, the sample was illuminated for some days and measured again, obtaining Seebeck values around -1 µV/K for compound **3a**, and around 2 µV/K for compound **3b**. For compound **3a** the most probable

Seebeck coefficient of the second sample was also measured after illumination, then the sample was kept in the dark for some days. A similar Seebeck coefficient was obtained around -1 μV/K, demonstrating the non-reversibility of the effect of light on the thermoelectric properties of the compound.

These results suggest sample degradation, possibly caused by the illumination of the sample by the white LEDs of a camera inside the STM. The measurements were repeated in the absence of light and the reported values of -2.7 and -0.4 μV/K were obtained for **3a** and **3b**, respectively, corresponding to the original BPS compounds avoiding degradation. A significant change was not observed in the conductance behavior.

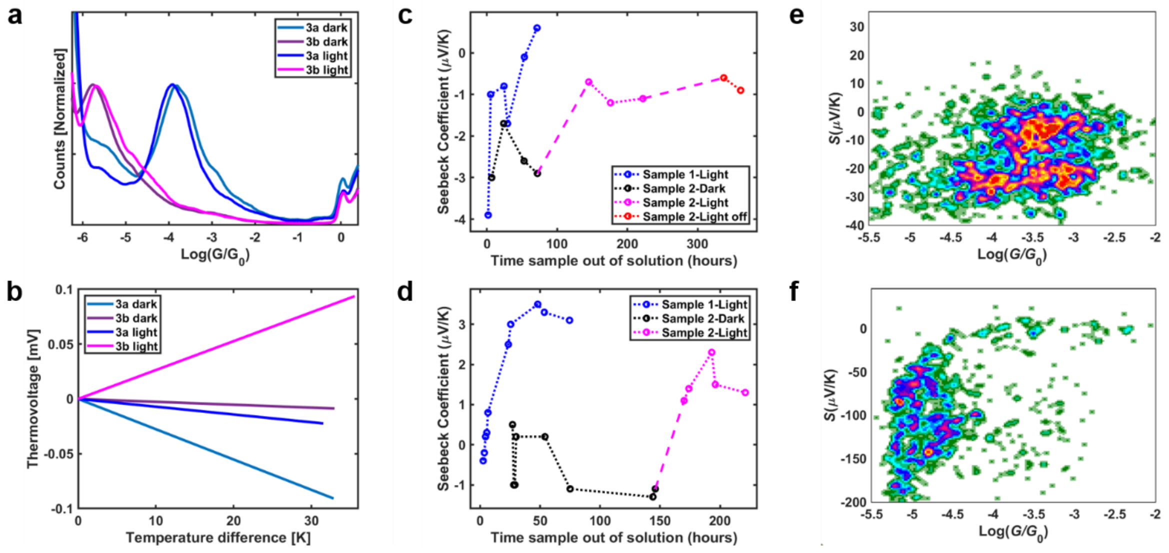

Figure S31. (a) Conductance histograms for compounds **3a** and **3b** both in the dark and under the white light of the camera. (b) Linear regressions of the temperature difference dependence of the thermovoltage measurements for both compounds in the dark and under light, without the high Seebeck coefficient data points. (c,d) Time dependence of the total Seebeck coefficient value for a sample measured under the light immediately after molecular deposition (Sample 1) and a sample measured in the dark for several days and then measured after some days of exposure to light (Sample 2) for compounds **3a** and (d) **3b**, respectively. For sample 2 of compound **3a** in (c), the Seebeck coefficient was also measured after some days in the dark following the days of exposure to light, demonstrating the non-reversibility of the effect. (e,f) Conductance vs. Seebeck coefficient plots of the high Seebeck coefficient data points around

two probable values: around -7 and -23 μV/K for compound **3a**, and around -75 and -117 μV/K for compound **3b**, respectively.

Given that both **3a** and **3b** were unstable on a gold surface under illumination, an XPS study was performed to understand this behavior.

C) XPS measurements

An XPS study was performed to understand the behavior of the BPS compounds (**3a** and **3b**) on a gold surface. A pre-annealed gold substrate (Arrandee) was incubated during 48 h in a 1 mM solution of **3a** and **3b** in DCM to enable self-assembled monolayer (SAM) formation. Examination of XPS data for the nitrogen 1s region is informative in studying this possible degradation. Illustrative XPS data for the nitrogen 1s region for **3a** and **3b** is shown in Figure S32. XPS characterization of a SAM of **3b** shows that upon illumination of the sample with a white LED during 48 h, only a peak at 399.8 eV is observed (Figure S32b). On the contrary, the XPS data for the powder shows two peaks at 399.8 eV, attributed to the amine, and at 401.5 eV, attributed to the positively charged nitrogen (N^+) (Figure S32a) [9]. These results confirm the degradation of **3b** when supported on a gold substrate by the conversion of the N^+ into a non-charged amine. The same results were obtained for compound **3a** (Figure S32c). It is noteworthy to indicate here that X-ray excitation in the analysis chamber of the XPS instrument causes the same degradation, as was observed in a SAM of **3a** without being illuminated previously; the relative intensity of the peak attributed to the N^+ moiety decreases with respect to the peak due to the amine in the powder (Figure S32d).

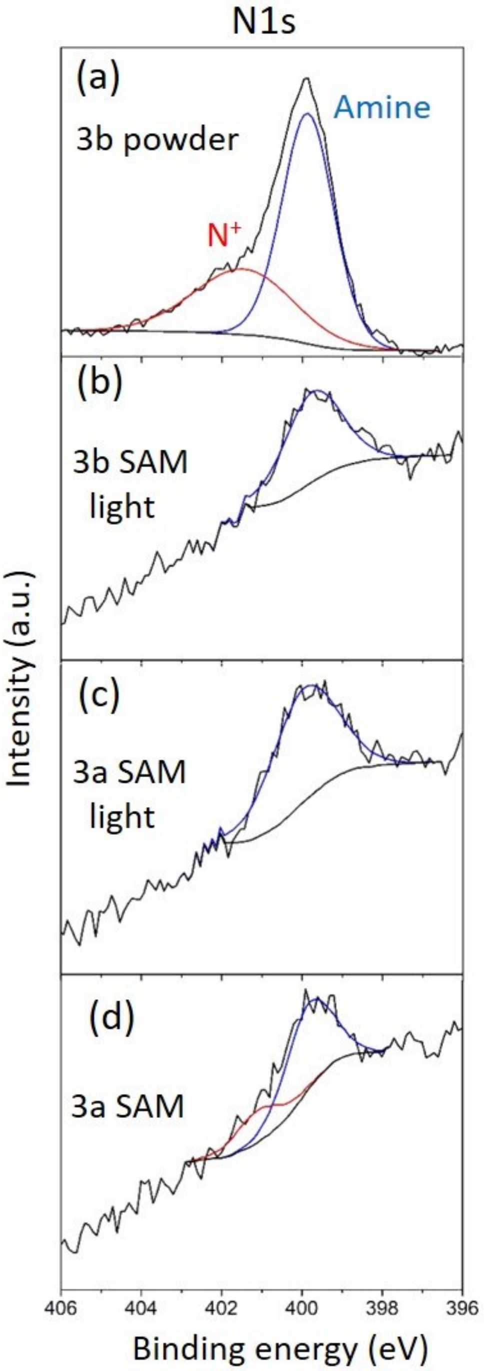

Figure S32. XPS spectra of the nitrogen 1s region for a powder sample of **3b** (a), a SAM of **3b** and **3a** under illumination (b) and (c); respectively and a SAM of **3a** without illumination (d) on a gold substrate.

D) Theory

Methodology

To study the electrical properties of the various molecules, the Hamiltonian matrices for each structure were obtained using density functional theory (DFT). The optimized molecular structure or ground-state geometry of the molecules were self-consistently obtained using the SIESTA[10] implementation of DFT. Next, the relaxed molecule was placed in a junction and relaxed again.

Generalized gradient approximation (GGA) of the exchange and correlation functional was used with the Perdew-Burke-Ernzerhof (PBE) parameterization and the following parameters: double-ζ (and single-ζ for gold Au) and a real space grid defined with an equivalent energy cut-off of 150 Rydberg (Ry). The geometry optimization for each structure was performed for the forces smaller than 0.02 eV/Å. The DFT obtained Hamiltonian of the relaxed junction was combined with the Gollum implementation of the non-equilibrium Green's function method to calculate the phase-coherent, electrical scattering properties of each junction, consisting of the source- (left) and drain- (right) leads and the scattering region (in between both leads). Gollum [11] was used to calculate the transmission coefficient $T(E)$ for electrons of energy E passing from the source to the drain. $T(E)$ can be described via the following relationship as in Eq. S2:

$$T(E) = trace(\Gamma_R(E) G^R(E) \Gamma_L(E) G^{R\dagger}(E)) \quad \textbf{(Eq. S2)}$$

With Eq. S3

$$\Gamma_{L,R}(E) = i(\Sigma_{L,R}(E) - \Sigma_{L,R}(E)^{-\dagger}) \quad \textbf{(Eq. S3)}$$

describing the level broadening due to the coupling between left (L) and right (R) electrodes and the central scattering region, $\sum_{L,R}(E)$ are the retarded self-energies associated with this coupling. Eq. S4

$$G^R = (ES - H - \Sigma_L - \Sigma_R)^{-1} \qquad \textbf{(Eq. S4)}$$

represents the retarded Green's function, where H is the Hamiltonian and S is the overlap matrix. The electrical conductance is calculated using the Landauer formula as follows Eq. S5:

$$G = G_0 L^0 = \frac{2e^2}{h} * \int_{-\infty}^{\infty} dE\, T(E) * \left(-\frac{\partial f}{\partial E}\right) \qquad \textbf{(Eq. S5)}$$

where $f(E,T) = \frac{1}{1+e^{\frac{E-E_F}{k_B T}}}$ is the Fermi-Dirac probability distribution function, T is the temperature, E_F is the Fermi energy, $G_0 = \frac{2e^2}{h}$ is the conductance quantum, e is the electron charge and h is Planck's constant. From Eq. S6:

$$L^n = \int_{-\infty}^{\infty} dE\left(E - E_f\right)^n * T(E) * \left(-\frac{\partial f}{\partial E}\right) \qquad \textbf{(Eq. S6)}$$

the Seebeck coefficient is obtained as:

$$S = \frac{-1}{eT}\frac{L^1}{L^0} = \frac{-1}{eT}\frac{\int_{-\infty}^{\infty} dE\left(E-E_f\right)*T(E)*\left(-\frac{\partial f}{\partial E}\right)}{\int_{-\infty}^{\infty} dE\, T(E)*\left(-\frac{\partial f}{\partial E}\right)} \qquad \textbf{(Eq. S7)}$$

Possible degradation products

Although the explicit structures of the **3a** and **3b** decomposition products are not known, their unusually high Seebeck coefficients and reasonable conductance values warranted a discussion as to what the compounds could be, as well as the origin of their physical behavior. The XPS data demonstrated that of the atoms investigated (oxygen, nitrogen, sulfur, carbon and gold), the most significant change occurred at the pyrrole nitrogen with its loss of charge, with no other identifiable changes for the remaining atoms, suggesting that much of the molecules remain unchanged. Therefore, it seems that an intramolecular electron transfer occurs during the irradiation of the donor-acceptor chromophore. Given that both pyrrole nitrogens are neutrally charged post-irradiation, this suggests a loss of the conjugative bond between the pyrrole units and the squaraine. Moreover, given the typical nature of this reaction, we focused on proposed structures that are compatible with the data available without attempting to explain the reaction mechanism in this study. Two potential structures were proposed that fit these criteria: the first is a reduction of the BPS alkene to an alkane, and the second is the formation of a dimer-like structure at the squaraine carbon, see Figures S33a and b. It can be noted that the data for the products from **3a** and **3b** are not consistent with the known photooxidative degradation of π-extended squaraines in solution.[12]

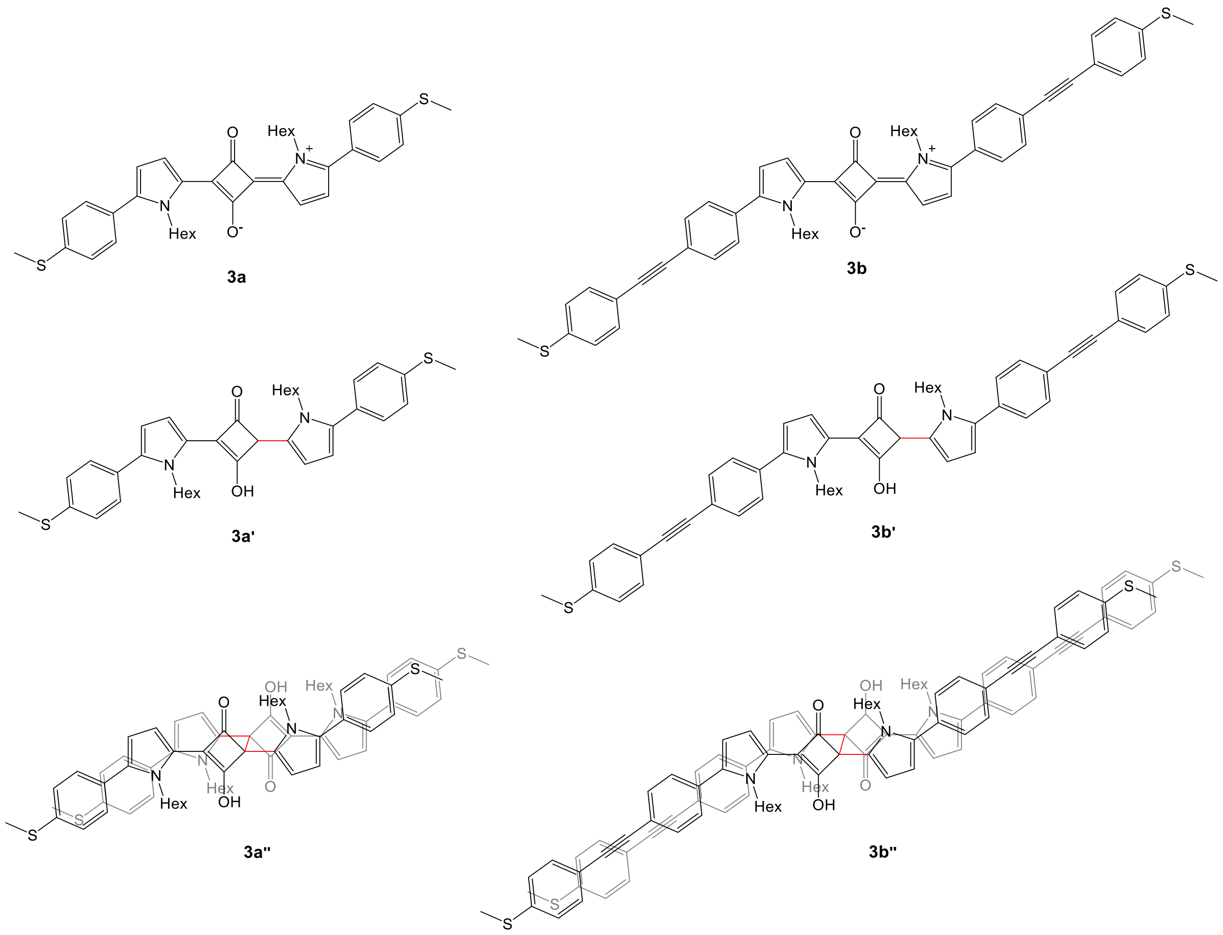

Figure S33. Proposed decomposition products of **3a** (left) and **3b** (right) at the top, in the middle the reduced structure denoted by **3a′** and **3b′** and at the bottom the structures denoted by **3a″** and **3b″**. The red bonds show the sites of the reduction and dimerization reactions.

Compounds 3a′ and 3b′

The first proposed degradation structure for compounds **3a** and **3b**, are **3a′** and **3b′** and are shown in Figures S33 middle row. Here, the inclusion of a hydrogen at the squaraine carbon results in the reduction of the alkene bond to give an alkane and a neutral pyrrole unit, thereby forming a skipped-conjugated system.[13] The calculated H-L gaps of 1.33 (**3a′**) and 1.27 (**3b′**) eV are smaller than those of the parent compounds **3a** and **3b**, with the HOMO and LUMO being localized to the core of the molecules including the hydrogen atom, as shown in Figures S34c and d. The uncorrected transmission shows complicated behavior and would need further

investigation that is beyond the scope of the present work. As a result of this behavior no match with the experimental Seebeck coefficient was found.

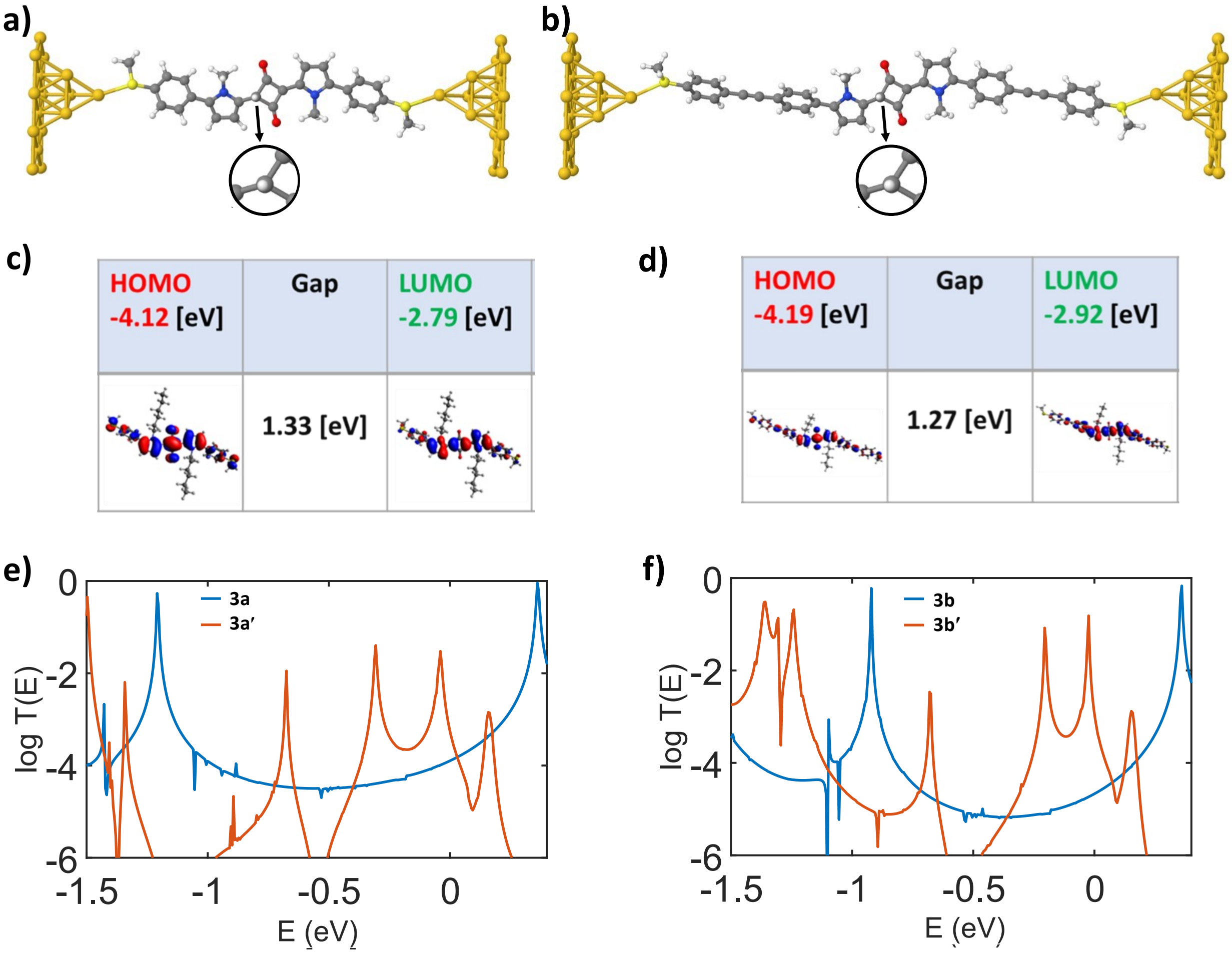

Figure S34. Structure in the junction of the degradation compounds **3a′** and **3b′** (with Me replacing hexyl) forming a covalent bond with hydrogen. (a) & (b), respectively. (c) & (d) Molecular orbitals of **3a′** and **3b′** left and right, respectively. (e) & (f) Electron transmission probability for **3a**, **3a′** and **3b**, **3b′**, respectively.

Compound 3a″ and 3b″

The second proposition is due to a covalent dimerization at the squaraine unit as shown in Figure S32 bottom row as **3a″** and **3b″**. The bond forms between the two carbon atoms of the squaraine resulting in reduction of the alkene between the squaraine and pyrrole to an alkane, as shown in Figure S35a. This results in a molecule with a smaller H-L gap of 0.1 eV, and a

HOMO delocalized over the entire conjugated backbone of the structure as shown in Figure S36c. The formation of such a molecule would result in a system with four thiomethyl anchor groups and therefore the possibility of three distinct junction configurations. The naming system for each contact geometry is described as follows: if contacted by thiomethyl contacting via the geminal (**3a″-g** or **3b″-g**) or two different vicinal (i: including the second squaraine or ii: excluding the second squaraine group; **3a″-v-i**, **3b″-v-i** and **3a″-v-ii**, **3b″-v-ii**, respectively) arms of the molecule, see Figure S35a. As each configuration gives rise to different behavior, we will examine each in turn.

Starting with the geminal configurations **3a″-g** or **3b″-g**, a clear DQI feature, which is not present in the parent molecules, is observed in the HOMO-LUMO gap (0.0 to -1.2 eV) by examining the transmission curve of this configuration. This feature results in conductance values of ca. -4.5 and -6 log(G/G_0), greater than, or similar to, that of the parent molecule, but at E_F = -0.36 eV, which is expected to give Seebeck coefficients of -60.2 and -90.6 μV/K for **3a″-g** or **3b″-g** respectively. These high values are within the range observed during measurements of the respective compounds (Figure S31e, f).

Regarding the first vicinal configuration **3a″-v-i** and **3b″-v-i**, this configuration also displays a DQI feature similar to the geminal configuration. However, in the vicinal case, the feature is shifted ca. 0.8 eV closer to the LUMO. Due to a Fano resonance,[14] there is an expected conductance of -6.0 log(G/G_0). For E_F = -0.36 eV, the Seebeck values are 10 and 20 μV/K, respectively, which are inconsistent with the high measured values.

Finally, the transmission curves of the second vicinal configuration **3a″-v-ii** and **3b″-v-ii** over a range of -0.5 – 0.0 eV behave similarly to those of the geminal configurations (**3a″-g** or **3b″-g**), but they lack the prominent DQI feature observed for either the geminal or first vicinal configurations, giving rise to conductance values of -4.0 and -6.3 log(G/G_0), respectively. For

E_F = -0.36 eV, the Seebeck values are -90.6 and -110.4 μV/K for **3a″-v-ii** and **3b″-v-ii** respectively, both of which are within the range observed during measurements of the respective compounds.

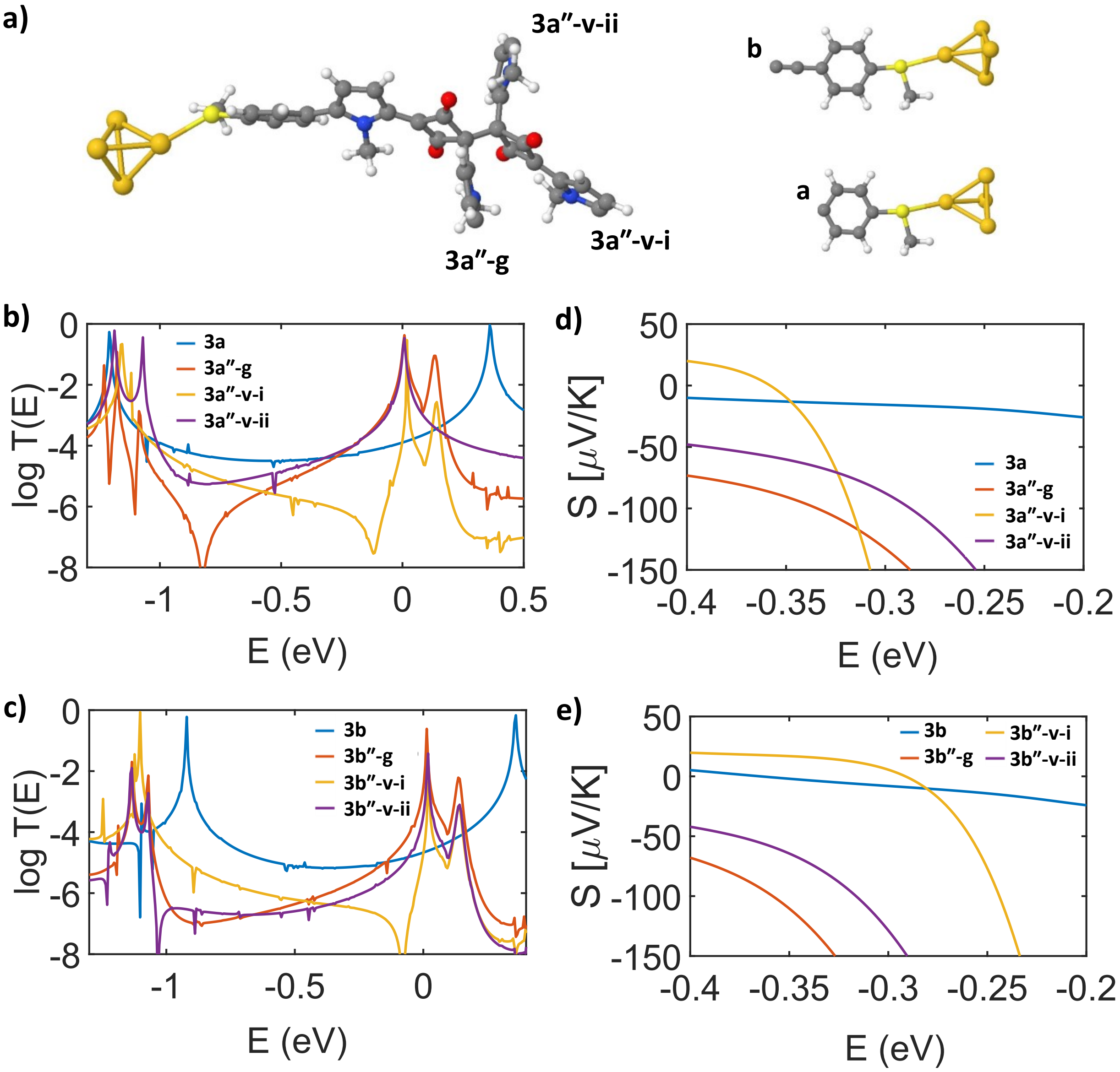

Figure S35. Structure in a junction, transmission and Seebeck coefficient for possible degradation structures **3a″** and **3b″** (a) Molecular structure of short configuration **3a″** in a junction with three different connections to the electrodes. Similar for **3b″**. (b) DFT transmission of **3a″** configurations. (c) DFT transmission of **3b″** configurations. (d) DFT Seebeck coefficient for **3a″**configurations. (e). DFT Seebeck coefficient for **3b″**configurations.

To further understand the origin of the DQI features in the H-L gap for **3a″** and **3b″**, we first examined the HOMO and LUMO orbitals and local density of states (LDOS), see Figures S36a, b and c. For both **3a″** and **3b″** both the HOMO and LUMO orbitals are delocalized along the geminal arms of the molecules with negligible contribution from the vicinal arms. Additionally, these molecules also display orbitals not directly coupled to either electrode, rather they are localized to the rest of the molecule, and these are described as "bound states". Due to the similarity in energy and proximity to the conductive frontier orbitals, these bound states form Fano resonances which can significantly impact the molecule's transport properties.[15–17] These bound states can reflect electrons to cause Fano resonances associated with sharp QI features observed in the transmission probability of certain molecules. The local density of state (LDOS) calculations for the HOMO and LUMO resonances of **3a″** and **3b″** conform with the orbitals of the isolated molecules as in Figure S36c. This confirms that the DQI observed for **3a″** and **3b″** can be attributed to Fano resonances.

From the experimental data, the decomposition products have similar conductance values to that of the parent molecules **3a** and **3b**, respectively. While all variants of **3a″** and **3b″** do not show the same conductance values as **3a** and **3b** by using the same E_F positioning as for the other molecules in this series, it is possible to find more optimal E_F values for these systems that give a better match with the measured data, e.g. E_F = -0.2 eV for **3b** and **3b″** gives more similar values of around 4.9 log(G/G_0), excluding **3b″-v-i**. Although it is not possible to conclusively state that either or both of the proposed decomposition molecules are responsible for the additional features measured in this study, the properties of these molecules certainly warrant further study beyond the scope of the present work.

a)

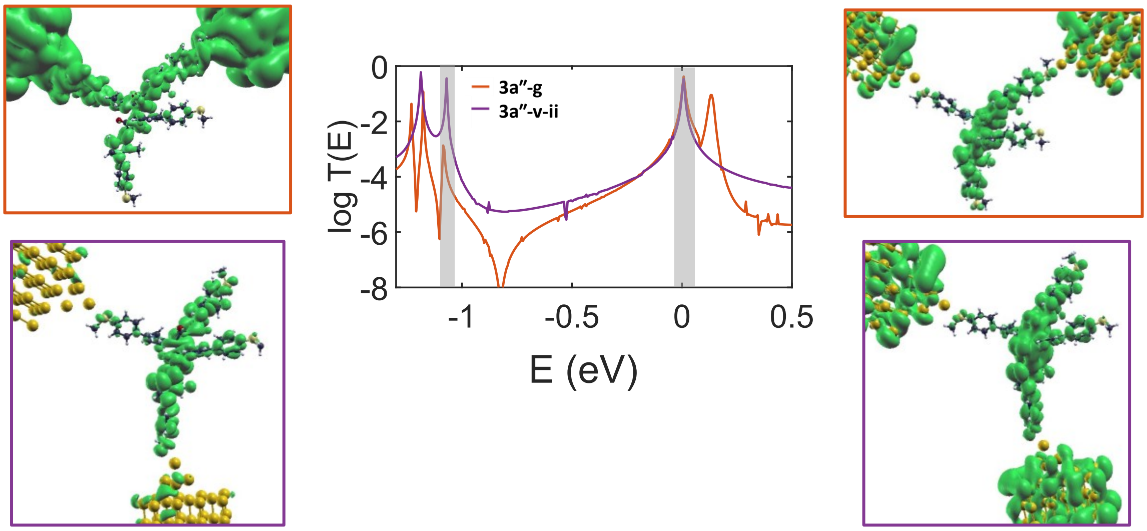

b)

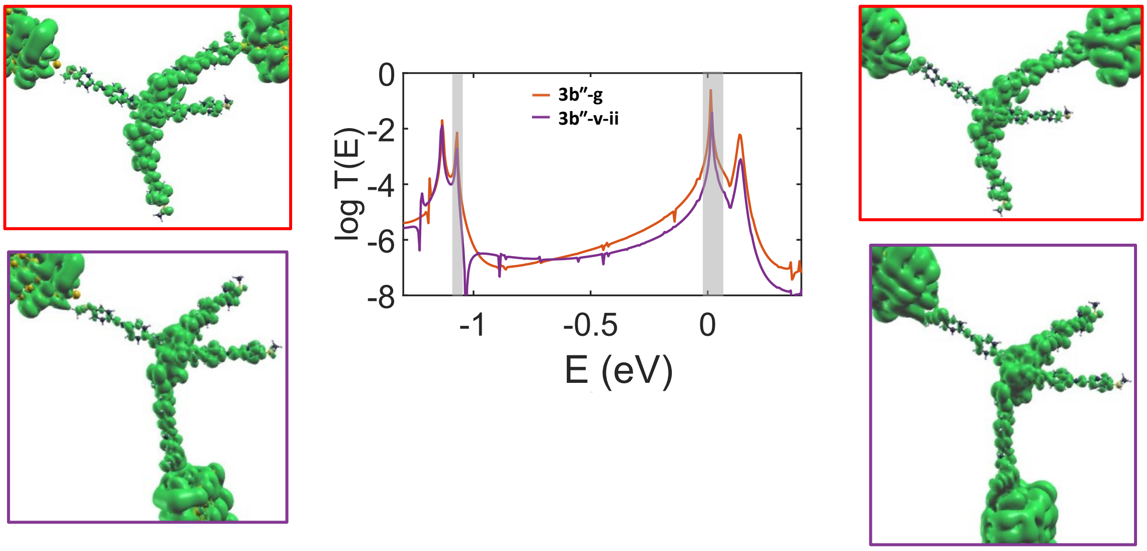

c)

HOMO -3.74 [eV]	Gap	LUMO -3.64 [eV]
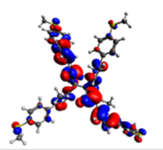	0.10 [eV]	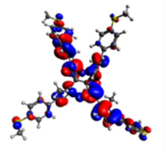

HOMO -3.86 [eV]	Gap	LUMO -3.77 [eV]
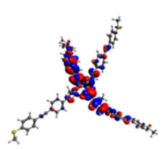	0.09 [eV]	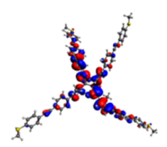

Figure S36. Local density of state calculations for **3a″** and **3b″**. (a) DFT transmission of **3a″-g** and **3a″-v-ii**, with the LDOS for the HOMO resonance (left) and the LUMO resonance (right). (b) DFT transmission of **3b″-g** and **3b″-v-ii**, with the LDOS for the HOMO resonance (left) and the LUMO resonance (right). Both indicate a bound state at the HOMO and LUMO resonances. (c) HOMO-LUMO orbitals for **3a″** and **3b″**, left and right respectively.

Thermoelectric properties of compounds 1a, 1b, 2a, 2b, 3a and 3b

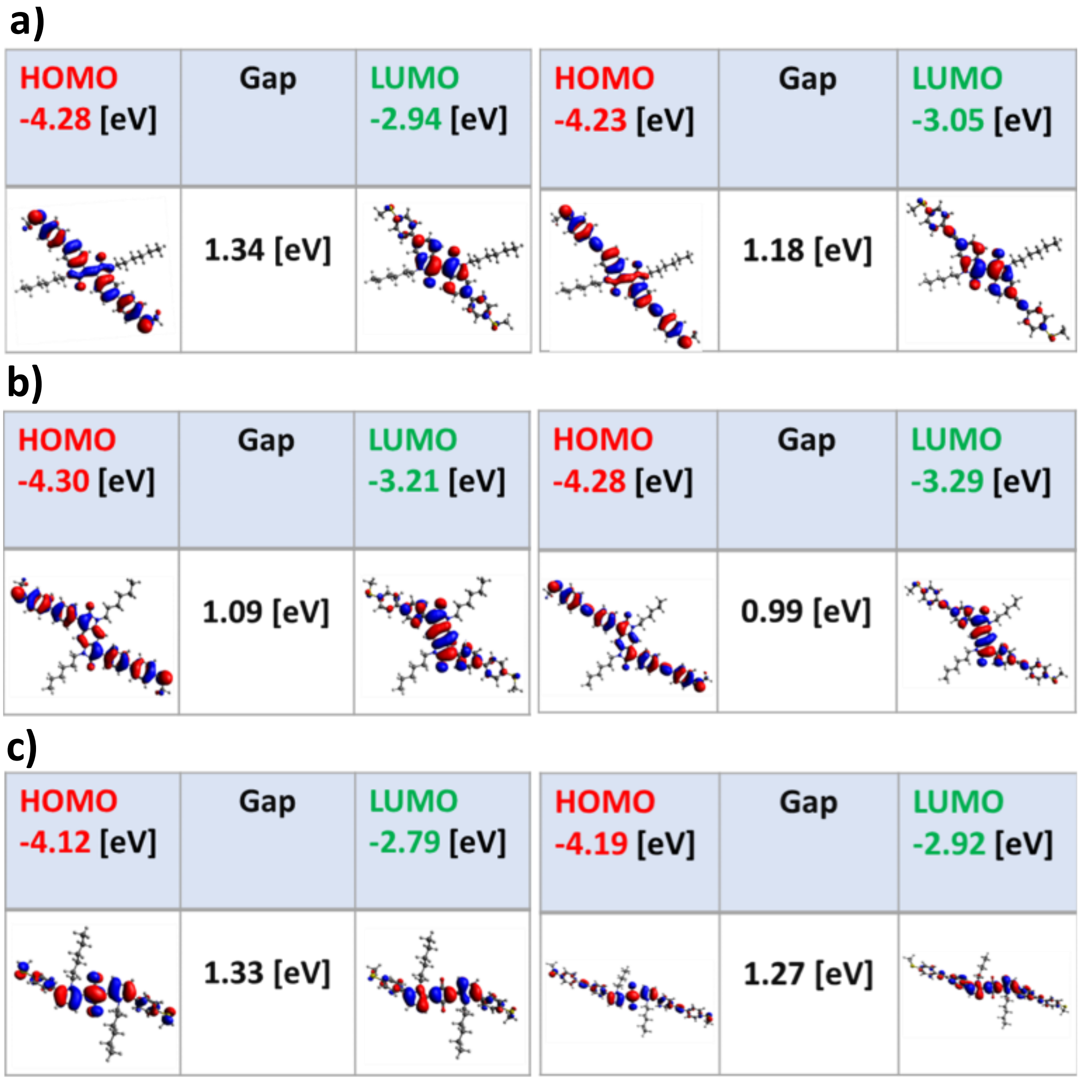

Figure S37a-c. The eigenvalues and orbitals of HOMO and LUMO of **1a**, **1b**, **2a**, **2b**, **3a**, and **3b**, respectively. The shorter structures (**a** series) are on the left and the longer analogues (**b** series) are on the right.

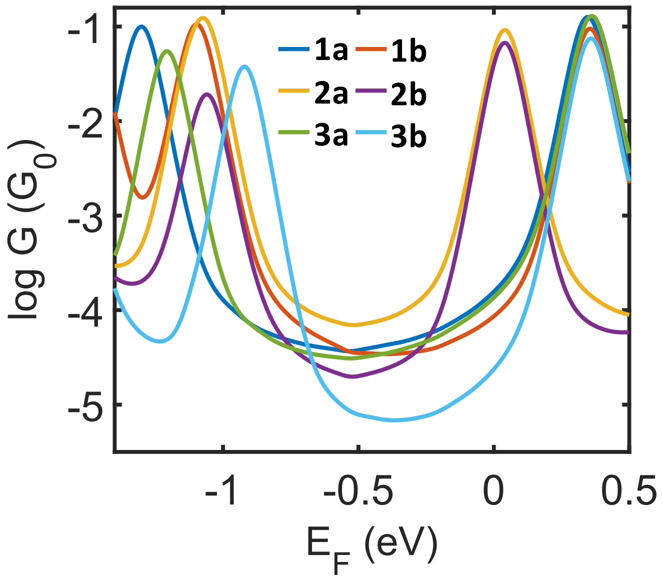

Figure S38. Conductance plots derived from theoretical electron transmission coefficient of compounds **1a**, **1b**, **2a**, **2b**, **3a** and **3b**.

Anchor Group dependence

To assess the impact of using different anchor groups to move the E_F closer to the LUMO resonance, analogs of **2a** and **2b** with pyridyl (**2a-Py** and **2b-Py**) and nitrile (**2a-CN** and **2b-CN**) anchors were modelled, see Figure S39.

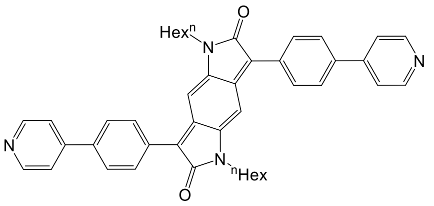

2a-Py

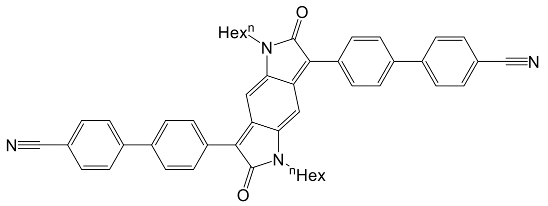

2a-CN

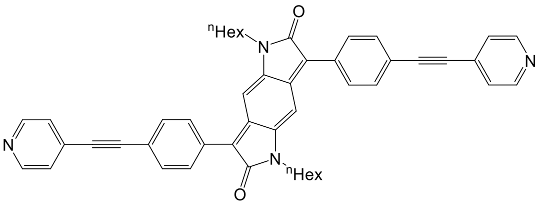

2b-Py

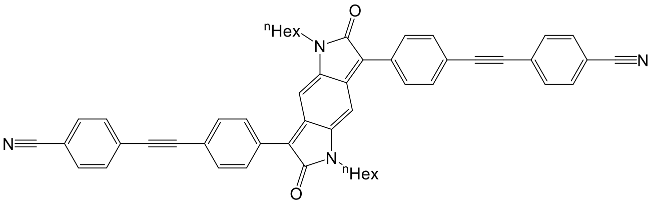

2b-CN

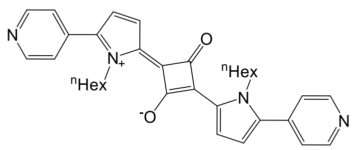

3a-Py

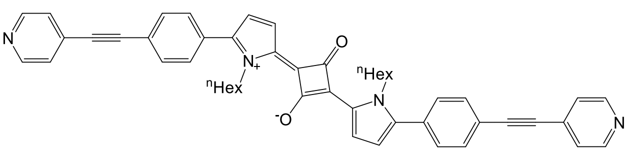

3b-Py

Figure S39. Schematic structures of proposed compounds **2a-Py**, **2b-Py**, **2a-CN** and **2b-CN**.

The new Py- and CN- anchored structures were placed in a junction and relaxed. Next the electron transmission probability was calculated and plotted in Figures S40a-b for **2a**, **2a-CN**, **2a-Py** and its longer Py analogs. In order to compare the effect, compounds **3a** and **3b** were also calculated with pyridyl anchors.

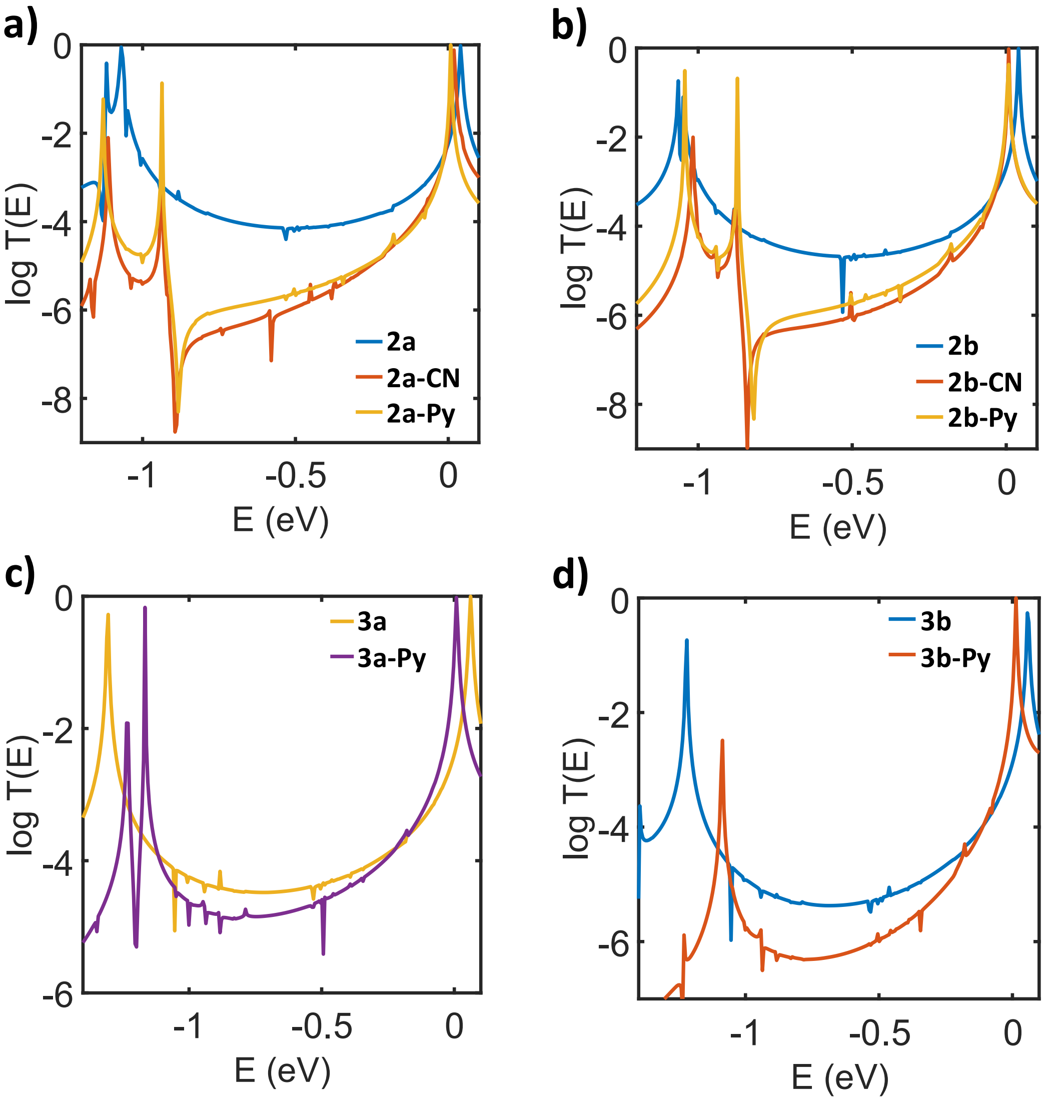

Figure S40. Calculated transmission curves for the pyridyl and nitrile analogues of **2a**, **2b**, **3a**, and **3b**. a) **2a**, **2a-CN**, **2a-Py**; b) **2b**, **2b-CN**, **2b-Py**; c) **3a** and **3a-Py**; d) **3b** and **3b-Py**.

For both compound **2a** and **2b** a lower transmission is the result of the two different anchor groups CN and Py. A Fano resonance is seen within the HOMO-LUMO gap. This results in a steeper gradient of the transmission around the Fermi energy. For **3a** and **3b** a smaller HOMO-LUMO gap is seen after changing the thioanisole anchor to a Py anchor. This again results in a steeper transmission gradient around the Fermi energy increasing the Seebeck coefficient.

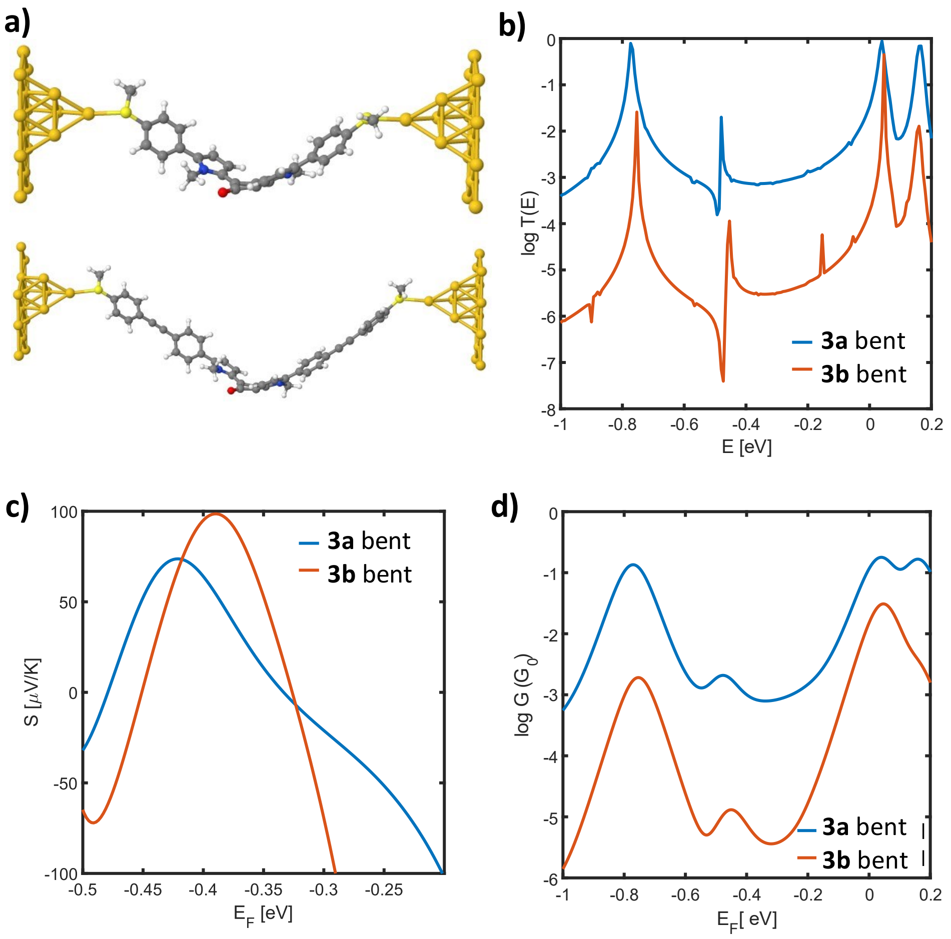

Figure S41. a) Bent **3a** and **3b** junctions top and bottom, respectively. b) Calculated transmission curves. c) Seebeck coefficient, and d) conductance of **3a** bent and **3b** bent which shows qualitative agreement with fully stretched junctions at the Fermi energy (E_F=0.36 eV).

References:

(1) Estrada, L. A.; Liu, D. Y.; Salazar, D. H.; Dyer, A. L.; Reynolds, J. R. Poly[Bis-EDOT-Isoindigo]: An Electroactive Polymer Applied to Electrochemical Supercapacitors. *Macromolecules* **2012**, *45* (20), 8211–8220. https://doi.org/10.1021/ma3016129.

(2) Wang, J.; Liu, S.; Chai, Z.; Chang, K.; Fang, M.; Han, M.; Wang, Y.; Li, S.; Han, H.; Li, Q.; Li, Z. Significantly Improved Performance of Dye-Sensitized Solar Cells by Optimizing Organic Dyes with Pyrrole as the Isolation Spacer and Utilizing Alkyl Chain Engineering. *J Mater Chem A Mater* **2018**, *6* (44), 22256–22265. https://doi.org/10.1039/C8TA06258G.

(3) Sun, Y. Q.; He, J.; Xu, Z.; Huang, G.; Zhou, X. P.; Zeller, M.; Hunter, A. D. Centripetal Molecules as Multifunctional Building Blocks for Coordination Networks. *Chemical Communications* **2007**, 45, 4779–4781. https://doi.org/10.1039/b709942h.

(4) Tang, P.; Furuya, T.; Ritter, T. Silver-Catalyzed Late-Stage Fluorination. *J Am Chem Soc* **2010**, *132* (34), 12150–12154. https://doi.org/10.1021/ja105834t.

(5) Krause, L.; Herbst-Irmer, R.; Sheldrick, G. M.; Stalke, D. Comparison of Silver and Molybdenum Microfocus X-Ray Sources for Single-Crystal Structure Determination. *J Appl Crystallogr* **2015**, *48* (1), 3–10. https://doi.org/10.1107/S1600576714022985.

(6) Sheldrick, G. M. SHELXT - Integrated Space-Group and Crystal-Structure Determination. *Acta Crystallogr A* **2015**, *71* (1), 3–8. https://doi.org/10.1107/S2053273314026370.

(7) Denisov, M.; Gorbunov, A.; Dmitriev, M. V.; Slepukhin, P.; Glushkov, V. Synthesis and Structure of Ferrocenol Esters. *Int J Org Chem (Irvine)* **2016**, *06* (02), 107–116. https://doi.org/10.4236/ijoc.2016.62012.

(8) Su, T. A.; Neupane, M.; Steigerwald, M. L.; Venkataraman, L.; Nuckolls, C. Chemical Principles of Single-Molecule Electronics. *Nat Rev Mater* **2016**, *1*, 16002. https://doi.org/10.1038/natrevmats.2016.2.

(9) Planje, I. J.; Davidson, R. J.; Vezzoli, A.; Daaoub, A.; Sangtarash, S.; Sadeghi, H.; Martín, S.; Cea, P.; Lambert, C. J.; Beeby, A.; Higgins, S. J.; Nichols, R. J. Selective Anchoring Groups for Molecular Electronic Junctions with ITO Electrodes. *ACS Sens* **2021**, *6* (2), 530–537. https://doi.org/10.1021/acssensors.0c02205.

(10) Soler, J. M.; Artacho, E.; Gale, J. D.; García, A.; Junquera, J.; Ordejón, P.; Sánchez-Portal, D. The Siesta Method for Ab Initio Order-N Materials Simulation. *J. Phys.: Condens. Matter* **2002**, *14*, 2745. https://doi.org/10.1088/0953-8984/14/11/302.

(11) Ferrer, J.; Lambert, C. J.; Garcia-Suarez, V. M.; Manrique, D. Zs.; Visontai, D.; Oroszlany, L.; Rodriguez-Ferradas, R.; Grace, I.; Bailey, S. W. D.; Gillemot, K.; Sadeghi, H.; Algharagholy, L. A. GOLLUM: A next-Generation Simulation Tool for Electron, Thermal and Spin Transport. *New J. Phys.* **2014**, *16*, 093029. https://doi.org/10.1088/1367-2630/16/9/093029.

(12) Rapozzi, V.; Beverina, L.; Salice, P.; Pagani, G. A.; Camerin, M.; Xodo, L. E. Photooxidation and Phototoxicity of π-Extended Squaraines. *J Med Chem* **2010**, *53* (5), 2188–2196. https://doi.org/10.1021/jm901727j.

(13) Salthouse, R. J.; Hurtado-Gallego, J.; Grace, I. M.; Davidson, R.; Alshammari, O.; Agraït, N.; Lambert, C. J.; Bryce, M. R. Electronic Conductance and Thermopower of Cross-Conjugated and Skipped-Conjugated Molecules in Single-Molecule Junctions. *Journal of Physical Chemistry C* **2023**, *127*, 13751–13758. https://doi.org/10.1021/acs.jpcc.3c00742.

(14) Miroshnichenko, A. E.; Flach, S.; Kivshar, Y. S. Fano Resonances in Nanoscale Structures. *Rev Mod Phys* **2010**, *82* (3), 2257–2298. https://doi.org/10.1103/RevModPhys.82.2257.

(15) Sam-Ang, P.; Reuter, M. G. Characterizing Destructive Quantum Interference in Electron Transport. *New J Phys* **2017**, *19* (5), 053002. https://doi.org/10.1088/1367-2630/aa6c23.

(16) Dhar, A.; Sen, D. Nonequilibrium Green's Function Formalism and the Problem of Bound States. *Phys Rev B Condens Matter Mater Phys* **2006**, *73* (8), 085119–14. https://doi.org/10.1103/PhysRevB.73.085119.

(17) Taylor, J.; Guo, H.; Wang, J. Ab Initio Modeling of Quantum Transport Properties of Molecular Electronic Devices. *Phys Rev B Condens Matter Mater Phys* **2001**, *63* (24), 245407. https://doi.org/10.1103/PhysRevB.63.245407.
